# In Silico Study, Synthesis, and Cytotoxic Activities of Porphyrin Derivatives

**DOI:** 10.3390/ph11010008

**Published:** 2018-01-20

**Authors:** Fransiska Kurniawan, Youhei Miura, Rahmana Emran Kartasasmita, Abdul Mutalib, Naoki Yoshioka, Daryono Hadi Tjahjono

**Affiliations:** 1 School of Pharmacy, Bandung Institute of Technology, Jalan Ganesha 10, Bandung 40132, Indonesia; fransiskakurniawan@yahoo.com (F.K.); kartasasmita@fa.itb.ac.id (R.E.K.); 2 Department of Applied Chemistry, Faculty of Science and Technology, Keio University, 3-14-1 Hiyoshi, Kohoku-ku, Yokohama 223-8522, Japan; y-miura@applc.keio.ac.jp (Y.M.); yoshioka@applc.keio.ac.jp (N.Y.); 3 Center for Radioisotope and Radiopharmaceutical Technology, National Nuclear Energy Agency (BATAN), Serpong, Tangerang 15310, Indonesia; mutalib@batan.go.id

**Keywords:** porphyrin derivative, molecular dynamics, synthesis, cytotoxicity, cancer cell lines

## Abstract

Five known porphyrins, 5,10,15,20-*tetrakis*(*p*-tolyl)porphyrin (TTP), 5,10,15,20-*tetrakis*(*p*-bromophenyl)porphyrin (TBrPP), 5,10,15,20-*tetrakis*(*p*-aminophenyl)porphyrin (TAPP), 5,10,15-*tris*(tolyl)-20-mono(*p*-nitrophenyl)porphyrin (TrTMNP), 5,10,15-*tris*(tolyl)-20-mono(*p*-aminophenyl)porphyrin (TrTMAP), and three novel porphyrin derivatives, 5,15-di-[*bis*(3,4-ethylcarboxymethylenoxy)phenyl]-10,20-di(*p*-tolyl)porphyrin (DBECPDTP), 5,10-di-[*bis*(3,4-ethylcarboxymethylenoxy)phenyl]-15,20-di-(methylpyrazole-4-yl)porphyrin (cDBECPDPzP), 5,15-di-[*bis*(3,4-ethylcarboxymethylenoxy)phenyl]-10,20-di-(methylpyrazole-4-yl)porphyrin (DBECPDPzP), were used to study their interaction with protein targets (in silico study), and were synthesized. Their cytotoxic activities against cancer cell lines were tested using 3-(4,5-dimetiltiazol-2-il)-2,5-difeniltetrazolium bromide (MTT) assay. The interaction of porphyrin derivatives with carbonic anhydrase IX (CAIX) and REV-ERBβ proteins were studied by molecular docking and molecular dynamic simulation. In silico study results reveal that DBECPDPzP and TrTMNP showed the highest binding interaction with REV- ERBβ and CAIX, respectively, and both complexes of DBECPDPzP-REV-ERBβ and TrTMNP-CAIX showed good and comparable stability during molecular dynamic simulation. The studied porphyrins have selective growth inhibition activities against tested cancer cells and are categorized as marginally active compounds based on their IC_50_.

## 1. Introduction

Cancer is a disease related to abnormal growth of cells. The primary cause of death in cancer cases is due to the cancer cells growing into the surrounding tissues and spreading (metastasis) to distant organs [1]. Based on the National Centre for Health Statistics, it was predicted that, in the USA, about 1,688,780 new cancer cases will appear in 2017 [2]. Early detection and treatment of cancer in early stages can increase the chance for curing the cancer and decrease the number of deaths significantly [3].

Several proteins that become important targets in cancer therapy are the ErbB receptor [4,5], HER-2/Neu [4], EGFR [4], tyrosin kinase [4], and carbonic anhydrase [6]. The description with respect to nine isozymes of carbonic anhydrase (CA I through IX) has been reported and it is known that CAs performs a variety of biological functions [6,7,8,9,10]. CA isozymes I through VII are expressed in normal tissue with various intensity and may be expressed in the malignant cell lines derived from the CA-expressing cells, but show no evidence for their direct relationships [6]. Carbonic anhydrase IX (CAIX) is an exception due to its expression, which is associated with tumorigenesis [6]. CAIX cannot be found in non-tumorigenic hybrid cells, but it is expressed in tumorigenic clones [11,12]. This enzyme was initially found in the surface of the HeLa cell line and its potential role to be a biomarker of cervical neoplasms has been investigated [13]. Another potential target for developing anticancer agents is REV-ERB, which is a protein in the nuclear receptor group containing most of the transcription factors. REV-ERB consists of REV-ERBα and REV-ERBβ, which regulate several physiological processes, including the circadian rhythm and metabolism [5]. Disruption of circadian rhythmicity is associated with the development of breast cancer based on epidemiological data [14,15,16,17,18] and the World Health Organization has classified shift-work associated with a disrupted circadian rhythm as a probable carcinogen [18,19]. Between these two types of REV-ERB, the REV-ERBβ is overexpressed in the cancer cell and its transcription is more than 95% from the total mRNA of REV-ERB [5].

The main treatment of cancer is surgery to remove the cancer cells from the normal tissue. However, this method only effective for the local cancer and it will be very difficult to handle metastatic cancer. Other methods are radiation therapy and chemotherapy. Each method can only kill a fraction of the cancer cells, thus, both methods are complementary [20]. A recent method applied to treat cancer is immune-based therapy (immunotherapy). This method can prevent the development of many cancers, but is not effective for all cancer-types [21].

Previous study showed that porphyrin derivatives bearing five-member rings of pyrazolium as *meso*-substituents have been successfully synthesized and it is known that they have strong interaction with DNA [22,23]. The porphyrin containing two carboxylate groups as *meso*-substituent, such as 3,4-*bis*(carboxymethyleneoxy)phenyl (3,4-BCP), has also been synthesized and reported to be used as a radiopharmaceutical ligand due to its selectivity to the melanoma and hepatoma cancer [24,25,26]. Furthermore, porphyrin molecules are known to have higher inhibition activity to cancer cells than to normal cells [27,28]. Thus, porphyrin derivatives bearing a combination of methyl pyrazole and 3,4-BCP as a *meso*-subtituent shows potential to be designed and developed as anticancer agent candidates.

The purpose of the present research was to design and synthesize novel porphyrin derivatives with methyl pyrazole and 3,4-BCP as a *meso*-subtituent, as well as their in silico study to obtain information on their interaction with CAIX and nuclear receptor REV-ERBβ which are associated with the cancer cells, and their cytotoxic effect on cancer cell lines. Five known porphyrins with more simple structure were also studied as comparison. The study was started with an in silico study to design and predict the interaction between porphyrin derivatives and the protein targets. The designed porphyrins were then synthesized and tested for their cytotoxicity against HeLa, WIDR, T47D, HepG2, and MCF-7 cancer cell lines. In addition, a comparison of the cytotoxic selectivity of porphyrin derivatives to cancer cells and normal cells was also evaluated by cytotoxicity testing against the Vero cell line.

## 2. Results

### 2.1. In Silico Study

All porphyrin derivatives were docked into carbonic anhydrase IX (PDB ID: 5FL6) and REV-ERBβ (PDB ID: 4N73) as targets. Table 1 shows the results of the docking simulation of the porphyrin derivatives.

**Figure 1:**
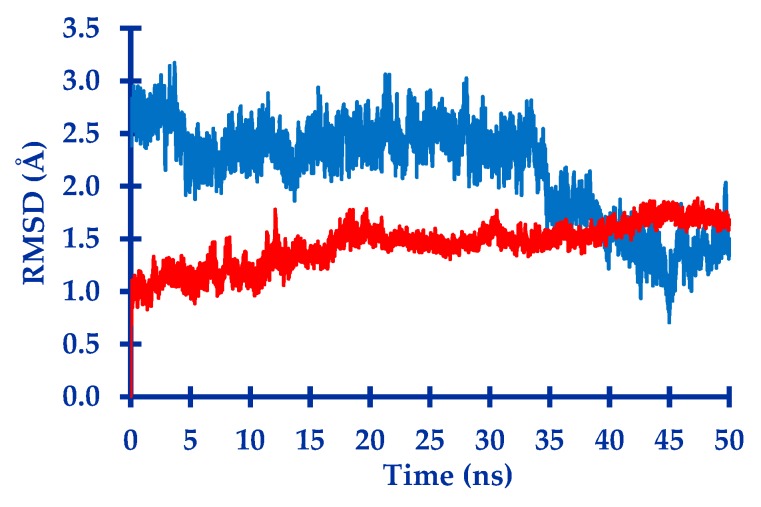
Plot of the RMSD value of the 5FL6-TrTMNP complex (red) and the 4N73-DBECPDPzP complex (blue) during the molecular dynamic simulation.

As summarized in Table 1, it showed that complexes 5FL6-TrTMNP and 4N73-DBECPDPzP have the largest negative binding energy and, hence, these complexes were predicted to have good binding interaction. Molecular dynamic simulation was performed for these two complexes for further study to provide structural, dynamic, and energetic information on their interaction. The stability of the complexes were then analyzed by the RMSD parameter (Figure 1).

**Figure 2:**
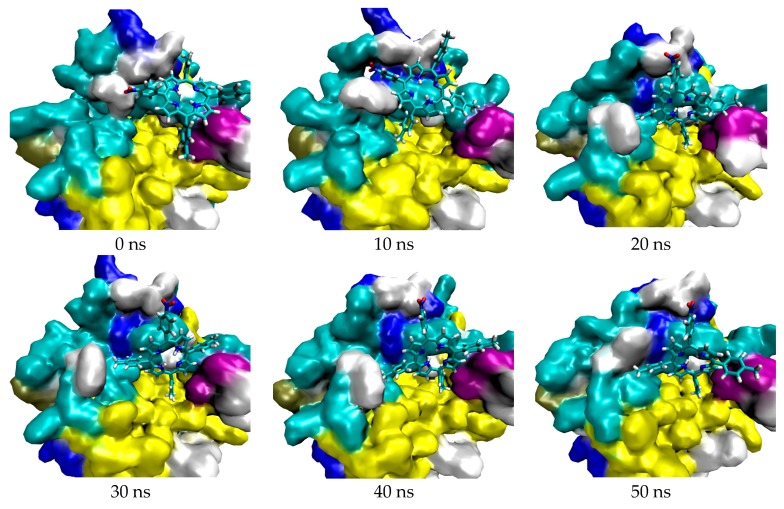
Trajectory of TrTMNP against 5FL6.

**Figure 3:**
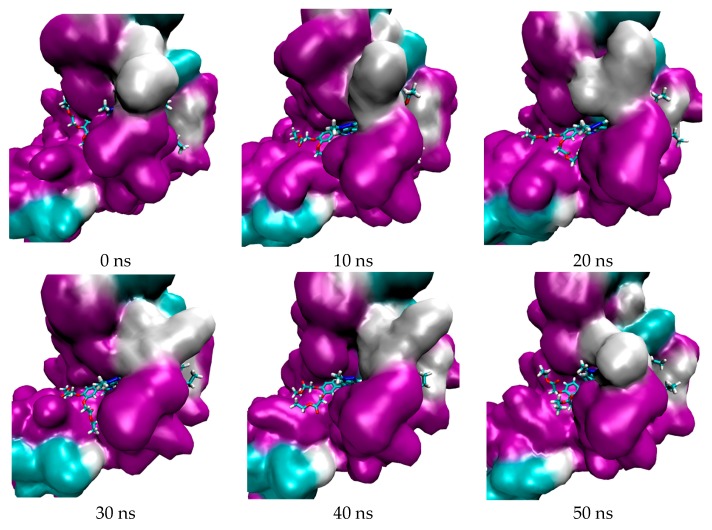
Trajectory of DBECPDPzP against 4N73.

The trajectory of both complexes were visualized to observe the position of the ligand and also to analyze their interaction. The visualization of their trajectory are shown in Figure 2 (for 5FL6-TrTMNP) and Figure 3 (for 4N73-DBECPDPzP).

After running the molecular dynamic simulation, the calculations of MM/PBSA were conducted to predict the value of ΔG_Bind_ (binding free energy), the contribution of each energy to the total calculated of binding free energy, and to evaluate the relative stability of each complex [29,30]. The results of MM/PBSA calculation for 5FL6-TrTMNP and 4N73-DBECPDPzP complexes is summarized in the Table 2.

### 2.2. Cytotoxicity Test

All of the porphyrin derivatives were evaluated for their cytotoxicity activities against several cancer cell lines and Vero cell line as a representative of normal cells. Table 3 shows the result of cytotoxicity test for porphyrin derivatives.

## 3. Discussion

### 3.1. In Silico Study

**Figure 4:**
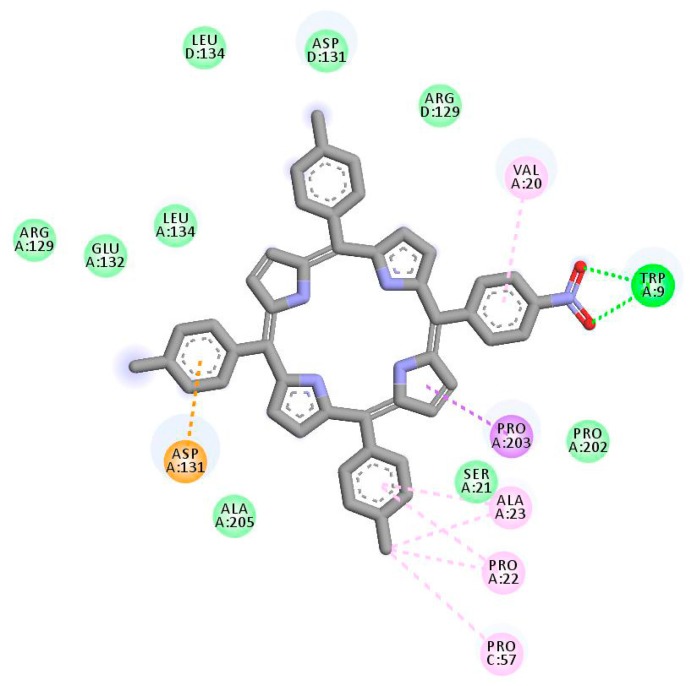
2D interaction between TrTMNP and CAIX.

**Figure 5:**
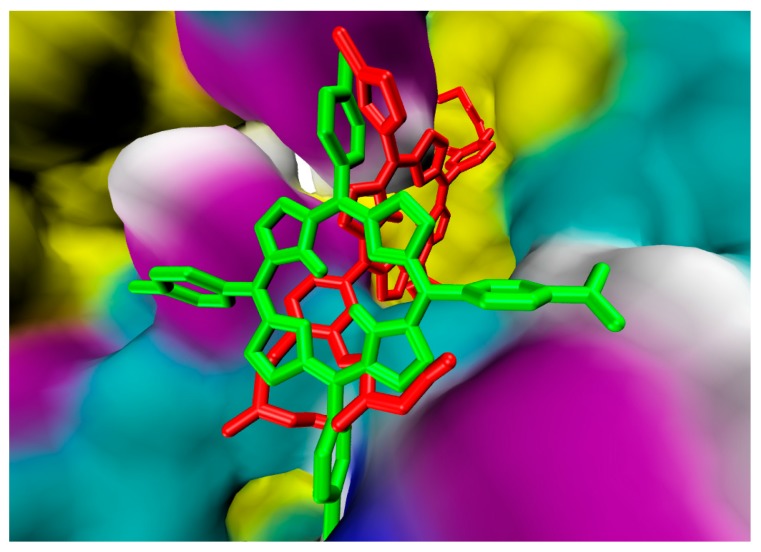
Overlay the docking pose of TrTMNP (green) and DBECPDPzP (red) in the binding site of 5FL6.

Each porphyrin derivative was docked to both targets and the complex having the largest negative binding energy was used to run molecular dynamic simulation and MM/PBSA calculation. Data in Table 1 shows that TrTMNP has the largest negative binding energy to the 5FL6 (CAIX target). All of the porphyrin derivatives have similar interaction to the CAIX (indicated by similar binding energy value). In general, the interaction of porphyrin with CAIX was dominated by electrostatic interaction with Arg129, Asp131, Glu132, and hydrophobic interaction with Arg64, Leu199, Arg62, Arg129, Pro22, and Ala23. Interaction of 5FL6-TrTMNP (Figure 4) consists of three hydrogen bonds (with Trp9), one electrostatic interaction (with Asp131), two π-σ interaction (with Asp131 and Pro203), three π-alkyl interactions (with Val20, Pro22, and Ala23), and three alkyl-alkyl interactions (with Pro22, Ala23, and Pro57). The docking result in Table 1 shows that DBECPDPzP has the lowest binding energy to the 5FL6. Visualization of the docking pose of TrTMNP and DBECPDPzP to 5FL6 (Figure 5) reveals that the position of the porphyrin ring shifted. In the case of DBECPDPzP, the shifting of the porphyrin core made different hydrophobic interactions and this phenomenon was predicted as the cause for increasing the binding energy value.

**Figure 6:**
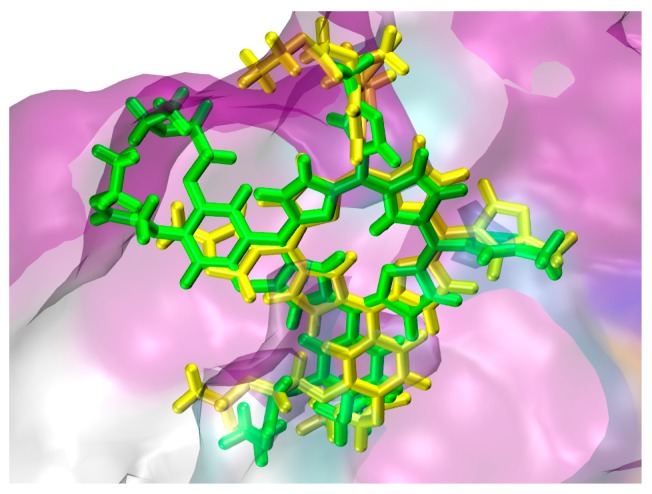
Overlay the docking pose of cDBECPDPzP (green) and DBECPDPzP (yellow) in the binding site of 4N73.

**Figure 7:**
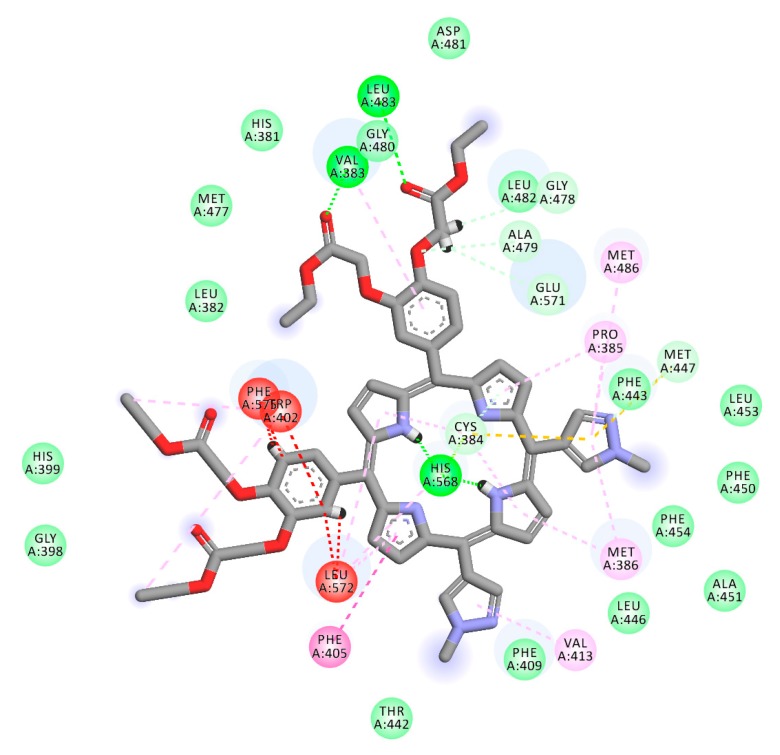
2D interaction between cDBECPDPzP and REV-ERBβ, which contains an unfavorable bond (red interaction) due to steric effects.

**Figure 8:**
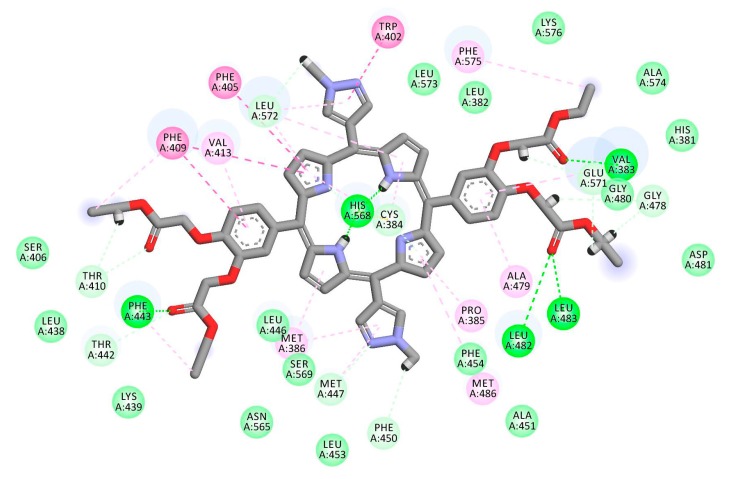
2D interaction between DBECPDPzP and REV-ERBβ.

The interaction between porphyrin derivatives and REV-ERBβ were dominated by hydrophobic interaction. Some of the porphyrin derivatives also show hydrogen bonds with the protein which contributes to stabilizing the complexes. Table 1 show that TAPP, cDBECPDPzP, and DBECPDTP have positive values of binding energy. Considering only these values, it was predicted that these three porphyrin derivatives would not have good interaction to the target. However, these porphyrin derivatives may have affinity to the target because of similar position in the binding site (e.g., Figure 6. The analysis of interactions mode between the protein and ligand (e.g., Figure 7) show that the positive values arose due to some unfavorable bonds between the protein and ligand which appear as a steric effect in the docking simulations performed by applying the principle of the rigid protein’s structure and flexible ligand. DBECPDPzP was predicted having the best interaction to the REV-ERBβ with the free binding energy of −29.75 kJ/mol. The interaction (Figure 8) was constructed by nineteen hydrogen bonds (with Val383, Cys384, Phe443, Gly478, Gly480, Leu482, Leu483, Thr410, Thr442, Met447, Phe450, His568, Glu571, and Leu572), four π-π interactions (with Trp402, Phe405, Phe409, and His568), and twelve π-alkyl interactions (with Val383, Cys384, Pro385, Met386, Phe409, Val413, Phe443, Met447, Ala479, Met486, Leu572, and Phe575). The best pose of DBECPDPzP to the REV-ERBβ receptor was selected for running the molecular dynamic simulation.

Figure 1 shows the RMSD of both complexes, which was used to evaluate their stability. The complexes are stable if the RMSD are constant and do not show large fluctuation. The 5FL6-TrTMNP complex shows better stability compared to 4N73-DBECPDPzP during the simulation. However, both complexes showed a minor movement with the RMSD of less than 3 Å [31].

Figure 2 and Figure 3 show the visualization of the complex during simulation, in which the TrTMNP pose shows significant changes, especially its nitrophenyl moeity. Comparing the pose at 0 ns to that at 50 ns, the pose of TrTMNP rotated nearly 90°. The CAIX structure binding site also encountered some changes to accommodate the proper interaction with TrTMNP. In the case of REV-ERBβ, no significant changes in the binding site were observed, but the DBECPDPzP structure could move inside the binding pocket. Likewise, no significant changes in the pose of DBECPDPzP and only the rotation in the side chain (BECP structure) were observed during the simulation, and the whole structure was translated to achieve a proper interaction.

MM/PBSA calculation was applied to predict more accurate on the binding free energy of protein-ligand complexes, and the results showed that their binding free energies were better than that observed in the docking simulation. Table 2 showed that ΔG_Bind_ of both complexes were dominated by ΔE_vdw_. The result confirms that the docking simulation gives proper information in which the interaction between porphyrin and the protein target was dominated by van der Waals interaction. The docking results showed some electrostatic interaction between the porphyrin derivatives and the target. However, the MM/PBSA calculation suggested that the ΔE_ele_ gives no significant effect to the ΔG_Bind_. Both complexes have good stability during molecular dynamic simulation, and 4N73-DBECPDPzP showed stronger binding interaction compared to that of 5FL6-TrTMNP, as expressed by the larger negative ΔG_Bind_.

### 3.2. Synthesis of Porphyrin Derivatives

**Scheme 1:**
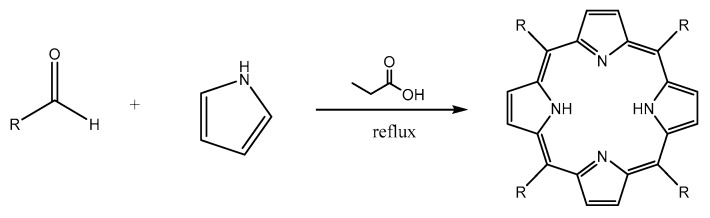
General Adler method to synthesize porphyrin derivatives.

All of porphyrin derivatives used in this study were synthesized by the Adler method (Scheme 1).

**Scheme 2:**
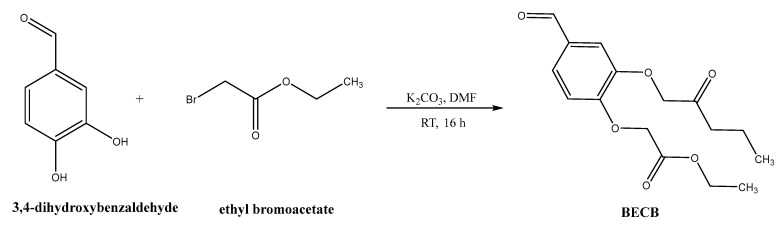
Synthetic route of BECB.

The synthesis of porphyrin derivatives were carried out by the Adler method because the purification and isolation of the product was relative simple [32], although the yield was relative low (usually below 20%). For the five known porphyrin derivatives, the aldehyde reagents were commercially available, so it can be directly reacted with pyrrole. However, in the case of three novel porphyrins containing *bis*-3,4 ethylcarboxymethylenoxyphenyl (BECP) as one of the meso-subtituents, the related aldehyde was not available commercially. Consequently, the aldehyde should be firstly synthesized. *Bis*-3,4 ethylcarboxymethylenoxy benzaldehyde (BECB) was prepared according to Scheme 2 [33].

The purpose of introducing the BECP as a meso-substituent was to increase the polarity of porphyrin derivatives. Increasing the polarity can contribute to better solubility in water. This is an important issue because most of anticancer drugs will be administered as an injection dosage form. Moreover, porphyrins containing BECP as the meso-substituent showed good affinity to the receptor as described in the in silico study, thus, it was predicted that these compounds be promising candidates for further in vitro cytotoxicity studies.

### 3.3. Cytotoxicity Test

From the data in Table 3, it can be concluded that all porphyrin derivatives were categorized as marginally-active compounds against tested cell line because they have IC_50_ in the range of 1 to 5000 μM [34], except for TrTMNP against T47D cell line, TrTMNP against the Vero cell line, as well as TTP against the Vero cell line. Comparing the value of IC_50_ against the cancer cell line and normal cell line (Vero cell line) can be used to proof that almost all synthesized porphyrins have higher anti-proliferation activities towards cancer cells compared to those of normal cells. Among the studied porphyrin derivatives, TTP and TrTMNP show better selective cytotoxic effect to the cancer cell compared to that of normal cell. Furthermore, if we refer the assumption that the compounds having IC_50_ less than 100 µM are potential candidates as anti-proliferative agents [35], the three novel porphyrins (cDBECPDPzP, DBECPDPzP, and DBECPDTP) also still have chance to be further investigated. These three novel porphyrins also show selective cytotoxic effects on cancer cells compared to the normal cells. Generally, increasing the polarity of the porphyrin by changing its meso-substituent can increase the cytotoxic effect on cancer cell lines, such as HepG2 and MCF-7. The result also showed that BECP as the meso-substituent of novel porphyrin derivatives increased the polarity of porphyrin derivatives and increase the cytotoxic effect on the cancer cell line.

CAIX was firstly identified as membrane-bonded protein on the surface of HeLa cells [36,37]. CAIX is a hypoxia-induced enzyme that is overexpressed in cancer cells [11]. The function of CAIX is to produce and manage the intracellular pH according to the proper environment for growth and survival of cancer cells. Thus, an inhibitor of CAIX can be a promising anticancer agent. Based on in silico study, all synthesized porphyrins have good binding interaction to CAIX. They also showed good cytotoxic activities against the HeLa cell line with an IC_50_ < 2 mM. DBECPDTP shows the highest anti-proliferative effect to the HeLa cell line with an IC_50_ of 201.7 μM. This is slightly lower than that of TrTMNP, which was predicted as the best ligand based on in silico study of the CAIX target.

Previous study showed that REV-ERBβ is dominant (more than 75%) in the MCF-7 cell line and the HepG2 cell line, whereas REV-ERBα is dominant in normal cells [5]. As observed in the in silico study, all porphyrin derivatives have interactions with REV-ERBβ, the in vitro test also confirmed that they have cytotoxicity activity to the MCF-7 and HepG2. DBECPDTP has the strongest cytotoxicity against MCF-7 cells with an IC_50_ of 42.1 μM, while cDBECPDPzP has the strongest cytotoxicity against HepG2 cells with an IC_50_ of 28.6 μM. Based on the above results, porphyrin derivatives which have low IC_50_ values are prospective compounds for further detailed pharmacological evaluation with respect to cellular accumulation, phototoxicity, as well as the mechanism of action.

## 4. Materials and Methods

### 4.1. In Silico Study

#### 4.1.1. Macromolecule Preparation

Macromolecules used in this study were carbonic anhydrase IX (CA IX) and nuclear receptor REV-ERBβ. Both receptor’s structures were downloaded from the Protein Data Bank with PDB ID 5FL6 and 4N73, respectively. The preparations of macromolecules were conducted by removing the water molecules and natural ligand, adding the polar hydrogen atoms, and calculating the Kollman charges.

#### 4.1.2. Ligand Preparation

**Figure 9:**
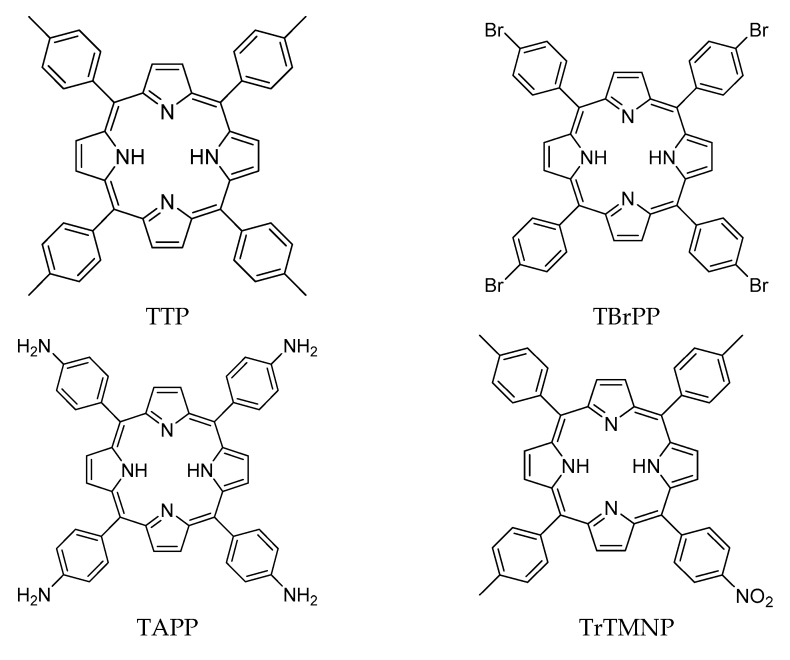

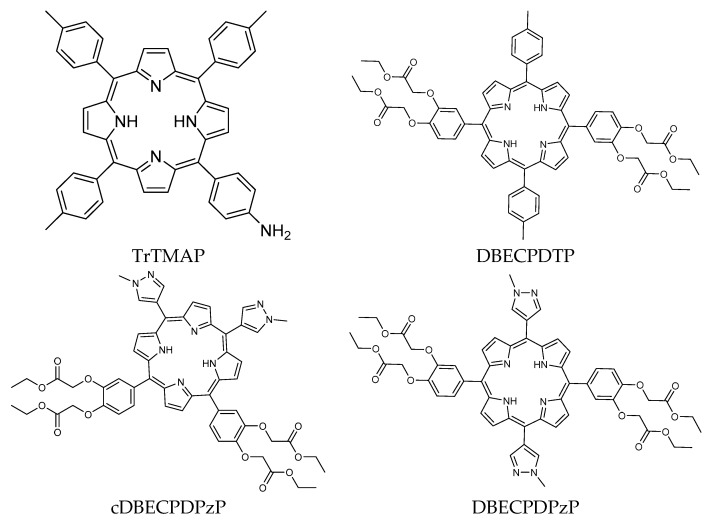
Structure of porphyrin derivatives.

Ligands used in the present study were eight porphyrin derivatives, of which five are known porphyrin derivatives and three are novel porphyrin derivatives (Figure 9). The ligand’s structures were prepared by GaussView 5.0.8 and optimized by Gaussian09 [38] using Density Functional Theory method with 6–31 basis sets. The optimized structure and the partial charges data were used as input for molecular docking studies.

#### 4.1.3. Molecular Docking Simulation

Molecular docking simulations were performed by AutoDock 4.2 with MGLTools 1.5.6 [39,40]. All ligands for this simulation were set as maximum torsion [41]. All simulations were performed using a grid box 64 × 60 × 60 points with 0.375 Å spacing. The Lamarckian Genetic Algorithm [40] was used with 100 conformations. All other docking parameters were set as default. Analysis of the results of the docking simulation was performed with VMD 1.9.2 [42] and Discovery Studio 2016 [43].

#### 4.1.4. Molecular Dynamic Simulation

Molecular dynamic simulations were performed for two complexes having the best value of binding free energy in each receptor. The simulations were performed by Gromacs2016 [44,45,46,47,48,49,50] and the analyses were performed with VMD 1.9.2 [42] and Discovery Studio 2016 [43]. AMBER99SB-ILDN force field [51] was used to parameterize the protein, while the ligand was parameterized using ACPYPE [52]. Long-range electrostatic force was determined by the Particle Mesh Ewald method [53,54]. Neutralization of the system was performed by adding Na^+^ and Cl^−^ ions. The cubic of TIP3P water model was used to solvate the system. The step of the simulation was included minimization, heating until 310 K, temperature equilibration (NVT), pressure equilibration (NPT), and production run with a 2 fs timestep for 50 ns. The stability of the system was verified by analysis of the energy, temperature, pressure, and root mean square deviation (RMSD).

#### 4.1.5. MM/PBSA Calculation

MM/PBSA calculation was performed by the g_mmpbsa package [55,56] integrated in Gromacs software. Polar desolvation energy was calculated with the Poisson-Boltzmann equation with a grid size of 0.5 Å. The dielectric constant of the solvent was set to 80 to represent water as the solvent [57,58]. Nonpolar contribution was determined by calculation of the solvent accessible surface area with the radii of the solvent as 1.4 Å. Binding free energy of the complex was determined based on 100 snapshots taken from 49 to 50 ns molecular dynamic simulation trajectories of the complex.

### 4.2. Synthesis of Porphyrin Derivatives

#### 4.2.1. Materials

Pyrrole (WAKO) and *p*-tolualdehyde (TCI) were distilled under reduce pressure before use. Phosphoryl chloride (WAKO), *p*-nitrobenzaldehyde (WAKO), *p*-bromobenzaldehyde (TCI), 1-methylpyrazole (TCI), 3,4-dihidroxybenzaldehyde (WAKO), ethyl bromoacetate (TCI), and SnCl_2_·2H_2_O (WAKO) were used as received. Other chemicals and solvents were of analytical grade and were purchased from Kanto Chemicals and WAKO. ^1^H-NMR and ^13^C-NMR spectra were recorded in the solvents indicated at 300 MHz (on JNM-LA 300 spectrometer) and 125 MHz (on Agilent Varian 500 MHz), respectively, and the chemical shift are reported in parts per million (ppm, δ). Mass spectra were recorded on a Brucker Ultraflex I MALDI-TOF LRMS or a Waters LCT-Premier ESI-LRMS.

#### 4.2.2. Procedure of Synthesis

##### Synthesis of 5,10,15,20-*tetrakis*(*p*-tolyl)porphyrin (TTP)

TTP was synthesized regarding reported method [32,59] with slightly modification. Distilled *p*-tolualdehyde (9.4 mL, 80 mmol) was added to the 300 mL of refluxing propionic acid. Pyrrole (5.6 mL, 80 mmol) was then added to the mixture and the mixture was refluxed for 2 h. After 2 h, the mixture was cooled to room temperature and filtered. The residue was then washed by hot water and methanol. The residue was dried in vacuum desiccator and purified by silica column chromatography using chloroform as eluent. Evaporation of the solvent resulted purple solid (4.47 g, 33.4% yield). ^1^H-NMR (CDCl_3_, 300 MHz): −2.79 ppm (s, 2H), 2.70 ppm (s, 12H), 7.54 ppm (d, *J* = 7.8 Hz, 8H), 8.09 ppm (d, *J* = 7.8 Hz, 8H), 8.84 ppm (s, 8H). ^13^C-NMR (CDCl_3_, 125 MHz): 21.5 ppm (CH_3_-tolyl), 120.1 ppm (C-*meso*), 127.3 ppm (C 3′,5′-tolyl), 127.4 ppm (C β-pyrrole), 134.5 ppm (C 2′,6′-tolyl), 134.5 ppm (C 4′-tolyl), 137.3 ppm (C 1′-tolyl), 139.3 ppm (C α-pyrrole). Melting point > 300 °C. UV/Vis (CH_2_Cl_2_): 420 nm (B-band), 516, 551, 591, and 647 nm (Q-band). MALDI-TOF LRMS: *m*/*z* = 671.007 [M + H]^+^ (exact mass = 671.318).

##### Synthesis of 5,10,15,20-*tetrakis*(*p*-bromophenyl)porphyrin(TBrPP)

TBrPP was synthesized regarding reported method [60] with slightly modification. *p*-bromobenzaldehyde (1.85 g, 10 mmol) was added to the 38 mL of refluxing propionic acid. Pyrrole (0.7 mL, 10 mmol) was then added to the mixture and the mixture was refluxed for 2 h. After 2 h, the mixture was cooled to room temperature and filtered. The residue was then washed by hot water and methanol. The residue was dried in vacuum desiccator and purified by silica column chromatography using chloroform as eluent. Evaporation of the solvent resulted dark purple solid (795.1 mg, 34.2% yield). ^1^H-NMR (CDCl_3_, 500 MHz): −2.86 ppm (s, 2H), 7.91 ppm (d, *J* = 8 Hz, 8H), 8.08 ppm (d, *J* = 8 Hz, 8H), 8.86 ppm (s, 8H). ^13^C-NMR (CDCl_3_, 125 MHz): 118.6 ppm (C-*meso*), 122.6 ppm (C 4′-bromophenyl), 130.0 ppm (C 2′,3′,5′,6′-bromophenyl), 131.5 ppm (C β-pyrrole), 135.8 ppm (C 1′-bromophenyl), 140.8 ppm (C α-pyrrole). Melting point > 300 °C. UV/Vis (CH_2_Cl_2_): 420 nm (B-band), 515, 549, 590, and 646 nm (Q-band). ESI LRMS: *m*/*z* = 927.317 [M + 2H]^2+^ (exact mass = 927.905).

##### Synthesis of 5,10,15,20-*tetrakis*(*p*-aminophenyl)porphyrin (TAPP)

TAPP was synthesized in accordance with the reported method [61] with slight modification. A solution of *p*-nitrobenzaldehyde (7.34 g, 48 mmol) and acetic anhydride (7.8 mL, 82 mmol) in 200 mL propionic acid was refluxed. Then pyrrole (3.4 mL, 48 mmol) was added dropwise as a solution in 6 mL propionic acid. The mixture was stirred for 30 min. The reaction was cooled to room temperature, filtered, and the collected residue was washed with hot water until the washings were colorless. Then the black residue was rinsed with methanol and dried in a vacuum desiccator. The residue was then mixed with 100 mL DMF and stirred for one hour at around 80 °C, cooled to room temperature, and the solution was stored in a refrigerator for 1.5–2 days. The mixture was filtered and the solid was washed with acetone until the washings were colorless. The dark purple product was dried under vacuum at room temperature to give 5,10,15,20-*tetrakis*(*p*-nitrophenyl)porphyrin (TNPP). The product (504 mg, 5.3% yield) was not soluble in common organic solvents and used for the next step without further purification. UV-VIS (tetrahydrofuran): 422 nm (B-band), 515, 549, 591, and 647 nm (Q-band).

A mixture of TNPP (500 mg, 0.63 mmol) and hydrochloric acid (12 M, 25 mL) was put in three-neck flask. A solution of SnCl_2_·2H_2_O (2.50 g, 11 mmol) in concentrated hydrochloric acid (5.4 mL) was added to the porphyrin mixture and the reaction mixture was heated using a water bath at 75–80 °C for 1.5 h. The hot-water bath was removed and replaced with a cold water bath, and then with an ice-water bath. The reaction was neutralized with sodium hydroxide solution. The mixture was then filtered and the black solid material was collected, washed twice with water. Extract the TAPP by using Soxhlet with acetone (250 mL). Evaporating the acetone afforded a shiny purple solid (322.7 mg, 76% yield). ^1^H-NMR (CDCl_3_, 300 MHz): −2.72 ppm (bs, 2H), 4.01 ppm (bs, 8H), 7.05 ppm (d, *J* = 8.4 Hz, 8H), 7.98 ppm (d, *J* = 8.4 Hz, 8H), 8.89 ppm (s, 8H). ^13^C-NMR (CDCl_3_, 125 MHz): 113.4 ppm (C-*meso*), 120.1 ppm (C 4′-aminophenyl), 130.9 ppm (C β-pyrrole), 132.7 ppm (C 2′,3′,5′,6′-aminophenyl), 135.7 ppm (C 1′-aminophenyl), 145.9 ppm (C α-pyrrole). Melting point > 370 °C. UV/Vis (CH_2_Cl_2_): 427 nm (B-band), 522, 563, 596, and 655 nm (Q-band). ESI LRMS: *m*/*z* = 675.314 [M + H]^+^ (exact mass = 675.299).

##### Synthesis of 5,10,15-*tris*(tolyl)-20-mono(p-nitrophenyl)porphyrin (TrTMNP)

TrTMNP was synthesized regarding reported method [62] with slightly modification. Propionic acid (200 mL) was put into a three-neck flask, then distilled pyrrole (3.4 mL, 48 mmol), *p*-tolualdehyde (7.2 mL, 72 mmol), and *p*-nitrobenzaldehyde (1.835 g, 12 mmol) were added. The mixture was stirred and refluxed for 2 h. After 2 h, the mixture was cooled to room temperature. The mixture was filtered using a glass filter and washed with hot water. The desired material was extracted from the glass filter using chloroform. The mixture was purified using column chromatography with silica as the stationary phase. The first column using chloroform as mobile phase to separate the dark material and all porphyrin compounds. A second column using chloroform/n-hexane = 5/1 was used to collect the desired porphyrin. TrTMNP was in the second band. Evaporating the solvent gave a purple solid (453.6 mg, 5.4% yield). ^1^H-NMR (CDCl_3_, 300 MHz): −2.77 ppm (s, 2H, broad), 2.72 ppm (s, 9H), 7.56 ppm (d, *J* = 8.1 Hz, 6H), 8.09 ppm (d, *J* = 7.8 Hz, 6H), 8.40 ppm (d, *J* = 8.7 Hz, 2H), 8.64 ppm (d, *J* = 8.4 Hz, 2H), 8.72 ppm (d, *J* = 5.4 Hz, 2H), 8.88 ppm (s, 4H), 8.91 ppm (d, *J* = 4.2 Hz, 2H). ^13^C-NMR (CDCl_3_, 125 MHz): 21.6 ppm (C CH_3_-tolyl), 116.3 ppm (C-15 *meso*), 120.7 ppm (C 10,20-*meso*), 121.2 ppm (C 5-*meso*), 121.8 ppm (C 3′,5′-nitrophenyl), 127.5 ppm (C 3′,5′-tolyl), 127.5 ppm (C β-pyrrole), 132.0 ppm (C β-pyrrole), 134.5 ppm (C 2′,6′-tolyl), 137.5 ppm (C 2′,6′-nitrophenyl), 137.5 ppm (C 4′-tolyl), 139.0 ppm (C 1′-tolyl), 139.0 ppm (C α-pyrrole), 147.6 ppm (C 4′-nitrophenyl), 149.4 ppm (C 1′-nitrophenyl). Melting point > 300 °C. UV/Vis (CH_2_Cl_2_): 425 nm (B-band), 517, 552, 591, and 647 nm (Q-band). MALDI-TOF LRMS: *m*/*z* = 702.731 [M + H]^+^ (exact mass = 702.287).

##### Synthesis of 5,10,15-*tris*(tolyl)-20-mono(p-aminophenyl)porphyrin (TrTMAP)

TrTMAP was synthesized according to the reported method [62] with slightly modification. A mixture of TrTMNP (150 mg, 0.214 mmol) and HCl (12 M, 20 mL) was put in a two-neck flask. A solution of SnCl_2_·2H_2_O (300 mg, 1.33 mmol) in concentrated HCl (5 mL) was added to the porphyrin mixture and the reaction mixture was heated using a water bath at 65–70 °C for two hours. The hot-water bath was removed and replaced with a cold water bath, and then with an ice-water bath. The reaction was neutralized with a sodium hydroxide solution. The mixture was then extracted with chloroform. The crude material was purified using column chromatography with chloroform/n-hexane = 3/1. Evaporating the solvent afforded a purple solid (50.7 mg, 35.3% yield). ^1^H-NMR (CDCl_3_, 300 MHz): −2.75 ppm (s, 2H), 2.71 ppm (s, 9H), 4.04 ppm (s, 2H), 7.06 ppm (d, 2H, *J* = 8.4 Hz), 7.55 ppm (d, 6H, *J* = 7.8 Hz), 8.00 ppm (d, 2H, *J* = 8.4 Hz), 8.09 ppm (d, 6H, *J* = 7.5 Hz), 8.85 ppm (s, 4H), 8.92 ppm (d, 2H, *J* = 4.8 Hz). ^13^C-NMR (CDCl_3_, 125 MHz): 21.5 ppm (C CH_3_-tolyl), 113.4 ppm (C 3′,5′-aminophenyl), 119.8 ppm (C 5-*meso*), 120.0 ppm (C 10,20-*meso*), 120.5 ppm (C 15-*meso*), 127.4 ppm (C 3′,5′-tolyl), 127.4 ppm (C β-pyrrole), 131.1 ppm (C β-pyrrole), 132.6 ppm (C 4′-aminophenyl), 134.5 ppm (C 2′,6′-tolyl), 135.6 ppm (C 2′,6′-aminophenyl), 137.3 ppm (C 4′-tolyl), 139.3 ppm (C 1′-tolyl), 139.4 ppm (C 1′-aminophenyl), 145.8 ppm (C α-pyrrole). Melting point > 300 °C. UV/Vis (CH_2_Cl_2_): 421 nm (B-band), 518, 554, 593, and 650 nm (Q-band). MALDI-TOF LRMS: *m*/*z* = 672.421 [M + H]^+^ (exact mass = 672.313).

##### Synthesis of *bis*(3,4-ethylcarboxymethylenoxy) Benzaldehyde (BECB)

BECB was synthesized in accordance with the reported method [33] with slight modification. 3,4-dihydroxybenzaldehyde (1.35 g, 9.78 mmol) and anhydrous potassium carbonate (5.4 g, 39.13 mmol) were added in a two-neck flask, then dissolved in dry DMF (20 mL) and stirred for 30 min until it changed into a yellow solution. The mixture was cooled to 0–5 °C using an ice bath. Ethyl bromo acetate (4.9 g, 29.34 mmol) was then added dropwise. The mixture was stirred at 0–5 °C for 30 min and then stirring continued at room temperature for 16 h. After 16 h, the mixture was extracted with brine and dichloromethane. The water phase was washed by dichloromethane, and the organic phase was then collected. The organic phase was extracted again with brine to remove DMF from the mixture. Sodium sulfate was added to the organic phase, filtered, and then evaporated. Purification of the crude product was performed by column chromatography for three times (using silica as stationary phase and ethyl acetate as mobile phase). The solvent was evaporated and the residue was kept under vacuum to obtain yellow oily product (1.3688 g, yield 45.2%).

^1^H-NMR (CDCl_3_, 300 MHz): 9.85 ppm (s, 1H), 7.49 ppm (dd, *J* = 1.8/8.4 Hz, 1H), 7.39 ppm (d, *J* = 1.5 Hz, 1H), 6.94 ppm (d, *J* = 8.4Hz, 1H), 4.81 ppm (s, 2H), 4.77 ppm (s, 2H), 4.28 ppm (q, *J* = 7.2 Hz, 4H), 4.12 ppm (q, *J* = 7.2 Hz, 4H), 1.32 ppm (t, *J* = 7.2 Hz, 3H), 1.28 ppm (t, *J* = 7.2 Hz, 3H).

##### Synthesis of 1-Methylpyrazole-4-carbaldehyde

1-Methylpyrazole-4-carbaldehyde was synthesized regarding reported method [22,63]. Dry dimethylformamide (34 mL) was cooled in an ice bath at 1–10 °C, then phosphoryl chloride (40 mL) was added dropwise. The reaction mixture was then stirred at room temperature for one hour. It was then heated to 90 °C and 10.2 mL of 1-methylpyrazole was added dropwise. The temperature was then increased to 90–95 °C for 1 h, 105–110 °C for 3 h, and 120–125 °C for 1 h. The still hot mixture was poured into about 500 mL of ice and the mixture was then diluted with 200 mL of water. The reaction mixture was left at room temperature overnight. Sodium bicarbonate was added until pH 5–6 and the solution was then extracted with dichloromethane. The organic phase was washed with brine and dried with sodium sulfate. The solvent was then evaporated and then the vacuum distillation was performed to obtain a yellowish liquid 1-methyl pyrazole-4-carbaldehyde (4.35 g, yield 32.22 %).

^1^H-NMR (CDCl_3_, 300 MHz): 9.85 ppm (s, 1H), 7.96 ppm (s, 1H), 7.90 ppm (s, 1H), 3.98 ppm (s, 3H).

##### Synthesis of 5,15-di-[*bis*(3,4-ethylcarboxymethylenoxy)phenyl]-10,20-di(*p*-tolyl)porphyrin (DBECPDTP)

*p*-tolualdehyde (0.1 mL, 1 mmol) and BECB (310 mg, 1 mmol) were added to 20 mL refluxing propionic acid. Pyrrole (0.14 mL, 2 mmol) was then added and the mixture was refluxed for 4 h. Propionic acid was removed by distillation under reduced pressure to give a dark residue. This was put into a minimum amount of dichloromethane and subjected to silica column chromatography. The elution with dichloromethane gave a major product on the second band (reddish). Re-column chromatography of this crude on longer silica column chromatography and elution with chloroform gave the major dark red product on the second band. Evaporation of the solvent afforded pure DBECPDTP as a purple solid (14.6 mg, yield 1.39%). ^1^H-NMR (CDCl_3_, 300 MHz): −2.84 ppm (bs, 2H), 1.13 ppm (t, *J* = 7.2 Hz, 6H), 1.40 ppm (t, *J* = 7.2, 6H), 2.70 ppm (s, 6H), 4.18 ppm (q, *J* = 7.2 Hz, 4H), 4.39 ppm (q, *J* = 7.2 Hz, 4H), 4.84 ppm (s, 4H), 5.01 ppm (s, 4H), 7.22 ppm (d, *J* = 8.7 Hz, 2H), 7.72 ppm (s, 2H), 7.77 ppm (d, *J* = 8.4 Hz, 2H), 8.84 ppm (s, 8H). ^13^C-NMR (CDCl_3_, 125 MHz): 14.1 ppm (C CH_3_-BECP), 14.3 ppm (C CH_3_-BECP), 21.5 ppm (C CH_3_-tolyl), 61.3 ppm (C CH_2_-BECP), 61.5 ppm (C CH_2_-BECP), 66.5 ppm (C methylene-BECP), 66.9 ppm (C methylene-BECP), 113.3 ppm (C 2′-BECP), 119.0 ppm (C 5′-BECP), 120.3 ppm (C *meso*), 121.4 ppm (C 6′-BECP), 127.4 ppm (C 3′,5′-tolyl), 128.7 ppm (C β-pyrrole), 134.5 ppm (C 1′-BECP), 135.4 ppm (C 2′,6′-BECP), 135.7 ppm (C 4′-tolyl), 136.4 ppm (C 1′-tolyl), 137.4 ppm (C α-pyrrole), 139.1 ppm (C 3′-BECP), 146.0 ppm (C 4′-BECP), 147.8 ppm (C α-pyrrole), 168.8 ppm (C carboxyl-BECP), 169.2 ppm (C carboxyl-BECP). UV/Vis (CH_2_Cl_2_): 421 nm (B-band), 517, 550, 591, and 651 nm (Q-band). MALDI-TOF LRMS: *m*/*z* = 1051.696 [M + H]^+^ (exact mass = 1051.413).

##### Synthesis of 5,10-di-[*bis*(3,4-ethylcarboxymethylenoxy)phenyl]-15,20-di-(methylpyrazole-4-yl)porphyrin (cDBECPDPzP)

1-Methylpyrazole-4-carbaldehyde (110 mg, 1 mmol) and BECB (310 mg, 1 mmol) were added to 20 mL refluxing propionic acid. Pyrrole (0.14 mL, 2 mmol) was then added and the mixture was refluxed for 4 h. Propionic acid was removed by distillation under reduced pressure to give a dark residue. This was taken into a minimum amount of dichloromethane and subjected to silica column chromatography. The elution with dichloromethane gave product on the second band (reddish). Re-column chromatography of this crude on longer silica column chromatography and elution with chloroform gave the major dark red product on the fourth band. Evaporation of the solvent afforded pure cDBECPDPzP as a purple solid (28.2 mg, yield 5.47%). ^1^H-NMR (CDCl_3_, 300 MHz): −2.78 ppm (bs, 2H), 1.16 ppm (t, *J* = 7.2 Hz, 6H), 1.41 ppm (t, *J* = 7.2 Hz, 6H), 4.19 ppm (q, *J* = 7.2 Hz, 4H), 4.33 ppm (s, 6H), 4.40 ppm (q, *J* = 7.2 Hz, 4H), 4.86 ppm (s, 4H), 5.02 ppm (s, 4H), 7.73–7.78 ppm (m, 6H), 8.18 ppm (s, 2H), 8.33 ppm (s, 2H), 8.83 ppm (s, 2H), 8.86 ppm (d, *J* = 4.8 Hz, 2H), 9.10 ppm (d, *J* = 4.8 Hz, 2H), 9.13 ppm (s, 2H). ^13^C-NMR (CDCl_3_, 125 MHz): 14.1 ppm (C CH_3_-BECP), 14.3 ppm (C CH_3_-BECP), 39.5 ppm (C CH_3_-pyrazolyl), 61.4 ppm (C CH_2_-BECP), 61.5 ppm (C CH_2_-BECP), 66.5 ppm (C methylene-BECP), 66.9 ppm (C methylene-BECP), 110.5 ppm (C 2′-BECP), 113.3 ppm (C 4′-pyrazolyl), 119.2 ppm (C 5′-BECP), 121.6 ppm (C 15,20-*meso*), 122.7 ppm (C 5,10-*meso*), 128.8 ppm (C 6′-BECP), 128.9 ppm (C 5′-pyrazolyl), 131.0 ppm (C β-pyrrole), 134.0 ppm (C 1′-BECP), 136.4 ppm (C 3′-pyrazolyl), 143.6 ppm (C 3′-BECP), 146.1 ppm (C 4′-BECP), 147.9 ppm (C α-pyrrole), 168.8 ppm (C carboxyl-BECP), 169.1 ppm (C carboxyl-BECP). UV/Vis (CH_2_Cl_2_): 422 nm (B-band), 520, 558, 595, and 653 nm (Q-band). MALDI-TOF LRMS: *m*/*z* = 1033.925 [M + 3H]^3+^ (exact mass = 1033.410).

##### Synthesis of 5,15-di-[*bis*(3,4-Ethylcarboxymethylenoxy)phenyl]-10,20-di-(methylpyrazole-4-yl)porphyrin (DBECPDPzP)

1-Methylpyrazole-4-carbaldehyde (110 mg, 1 mmol) and BECB (310 mg, 1 mmol) were added to 20 mL refluxing propionic acid. Pyrrole (0.14 mL, 2 mmol) was then added and the mixture was refluxed for 4 h. Propionic acid was removed by distillation under reduced pressure to give a dark residue. This was taken into a minimum amount of dichloromethane and subjected to silica column chromatography. The elution with dichloromethane gave product on the second band (reddish). Re-column chromatography of this crude on longer silica column chromatography and elution with chloroform gave the major dark red product on the third band. Evaporation of the solvent afforded pure DBECPDPzP as purple solid (12.5 mg, yield 2.42%). ^1^H-NMR (CDCl_3_, 300 MHz): −2.77 ppm (bs, 2H), 1.17 ppm (t, *J* = 7.2 Hz, 6H), 1.42 ppm (t, *J* = 7.2 Hz, 6H), 4.21 ppm (q, *J* = 7.2 Hz, 4H), 4.34 ppm (s, 6H), 4.42 ppm (q, *J* = 7.2 Hz, 4H), 4.88 ppm (s, 4H), 5.03 ppm (s, 4H), 7.50–7.60 ppm (m, 2H), 7.70–7.80 ppm (m, 4H), 8.18 ppm (s, 2H), 8.33 ppm (s, 2H), 8.88 ppm (d, *J* = 4.8 Hz, 4H), 9.10 ppm (d, *J* = 4.8 Hz, 4H). ^13^C-NMR (CDCl_3_, 125 MHz): 14.1 ppm (C CH_3_-BECP), 14.3 ppm (C CH_3_-BECP), 39.5 ppm (C CH_3_-pyrazolyl), 61.4 ppm (C CH_2_-BECP), 61.5 ppm (C CH_2_-BECP), 66.5 ppm (C methylene-BECP), 66.9 ppm (C methylene-BECP), 110.0 ppm (C 2′-BECP), 110.7 ppm (C 5′-BECP), 113.3 ppm (C 4′-pyrazolyl), 119.1 ppm (C 5,15-*meso*), 121.6 ppm (C 10,20-*meso*), 122.8 ppm (C 6′-BECP), 128.8 ppm (C 5′-pyrazolyl), 131.0 ppm (C β-pyrrole), 134.1 ppm (C 1′-BECP), 136.3 ppm (C 3′-pyrazolyl), 143.7 ppm (C 3′-BECP), 146.1 ppm (C 4′-BECP), 147.9 ppm (C α-pyrrole), 168.8 ppm (C carboxyl-BECP), 169.1 ppm (C carboxyl-BECP). UV/Vis (CH_2_Cl_2_): 422 nm (B-band), 520, 558, 595, and 653 nm (Q-band). MALDI-TOF LRMS: *m*/*z* = 1034.477 [M + 4H]^4+^ (exact mass = 1034.417).

### 4.3. Cytotoxicity Test

#### 4.3.1. Materials

The human epithelioid cervix carcinoma cell line (HeLa), human ductal breast epithelial tumor cell line (T47D), Michigan Cancer Foundation-7 cell line (MCF-7), human colon carcinoma cell line (WIDR), hepatoblastoma-derived cell line (HepG2), and Vero cell line (derived from kidney of African green monkey) were purchased from ATCC by the Faculty of Medicine Gadjah Mada University. The cell lines were cultured in Dulbecco’s Modified Eagle Medium (DMEM, Gibco), except for Vero cell line, which was cultured in Medium-199 (M-199, Gibco). Other chemicals, i.e., potassium dihydrogen phosphate, sodium chloride, potassium chloride, sodium hydrogen phosphate, dimethyl sulfoxide, sodium dodecyl sulphate, HEPES, sodium bicarbonate, and 3-(4,5-dimetiltiazol-2-il)-2,5-difeniltetrazolium bromide (MTT) were purchased from Sigma-Aldrich. Fetal bovine serum, trypsin-EDTA 0.25%, and penicillin-streptomycin were purchased from Gibco, whereas Amphotericin B was purchased from Caisson. Phosphate-buffered saline (PBS) was obtained by mixing 0.2 g of KH_2_PO_4_, 8 g of NaCl, 0.2 g of KCl, 1.15 g of Na_2_HPO_4_, and 1 L of distilled water.

#### 4.3.2. Procedure

Confluent cells in the Petri dish was harvested by removing the growth media in Petri dish and adding 1 mL of trypsin-EDTA 0.025%. Then the cells was incubated at incubator 37 °C, 5% CO_2_ for about three minutes. The suspension of cells was put in centrifuge tube, 2 mL of growth media was added with 7 mL of PBS. The mixture was centrifuged for 10 min at 1200 rpm. The supernatant was separated and 2–3 mL of growth media was added to the pellet cell for counting cell. The cell suspense was cultured in 96-well plates (10,000 cells/well). After 24 h, the porphyrin derivative compounds with series concentration were added to treat the cell cultures for another 24 h. The cells were treated by MTT solution for four hours at incubator (37 °C, 5% CO_2_) and the reaction was stopped by stopper solution (SDS 10% HCl). The plate was incubated at room temperature in dark condition for 24 h. The living cells were counted by reading the absorbance at 595 nm. The corresponding IC_50_ values were calculated using non-linear regression analysis (GraphPad Prism 7.0.3). Each test was run in triplicate.

## 5. Conclusions

In silico study confirmed that DBECPDPzP has good interaction with the REV-ERβ receptor with a binding free energy of −276.46 kJ/mol, while TrTMNP showed appropriate interaction toward CAIX with a binding free energy of −15.50 kJ/mol. Five known porphyrin derivatives and three novel porphyrin derivatives have been successfully synthesized by the Alder method and their structures were confirmed. Cytotoxicity test against five cancer cell lines (HeLa, WIDR, T47D, MCF-7, HepG2) and the normal cell (Vero cell line) revealed that all studied porphyrins are categorized as marginally-active compounds with IC_50_ lower than 1.5 mM, except for TrTMNP against T47D cell line. It was observed that all of the porphyrins showed a selective anti-proliferation activity to cancer cells than to normal cells, except for cDBECPDPzP and DBECPDPzP to the HeLa cell line. The results obtained proved the necessity for further detailed biological investigation for studied porphyrins that showed low IC_50_.

## Figures and Tables

**Table 1:** Binding energy of porphyrin to protein targets.

Porphyrin Ligand	Binding Energy (kJ/mol)	
	5FL6	4N73
TTP	−26.82	−22.84
TBrPP	−29.00	−16.11
TAPP	−21.30	179.37
TrTMNP	−30.08	−15.69
TrTMAP	−28.66	−23.81
cDBECPDPzP	−22.34	36.15
DBECPDPzP	−16.90	−29.75
DBECPDTP	−18.87	224.26

**Table 2:** The binding free energies and their corresponding components of porphyrin bound to related protein targets.

Complex	ΔE_vdw_ (kJ/mol)	ΔE_ele_ (kJ/mol)	ΔG_PB_ (kJ/mol)	ΔG_NP_ (kJ/mol)	ΔG_Bind_ (kJ/mol)
5FL6-TrTMNP	−53.49	−9.39	53.51	−6.13	−15.50
4N73-DBECPDPzP	−477.91	−42.45	282.05	−38.15	−276.46

Note: ΔE_vdw_ = van der Waals contribution, ΔE_ele_ = electrostatic contribution, ΔG_PB_ = polar contribution of desolvation, ΔG_NP_ = non-polar contribution of desolvation.

**Table 3:** Cytotoxicity (IC_50_) of porphyrin derivatives against cell lines.

Porphyrins	IC_50_ (μM) ± SD					
	HeLa	WIDR	HepG2	T47D	MCF-7	Vero
TTP	714.6 ± 1.1	969.8 ± 1.2	1120.0 ± 1.3	1241.0 ± 1.2	1305.0 ± 1.3	15,612.0 ± 1.6
TBrPP	595.6 ± 1.3	772.7 ± 1.3	882.7 ± 1.2	443.9 ± 1.1	870.6 ± 1.3	1176.0 ± 1.1
TAPP	1007.0 ± 1.1	205.1 ± 1.2	36.0 ± 1.2	332.6 ± 1.2	262.2 ± 1.3	2264.0 ± 1.2
TrTMNP	226.1 ± 1.1	897.5 ± 1.1	146.6 ± 1.2	5372.0 ± 1.4	505.2 ± 1.2	10,079.0 ± 1.2
TrTMAP	593.1 ± 1.2	835.4 ± 1.3	106.7 ± 1.3	1099.0 ± 1.3	328.4 ± 1.4	4784.0 ± 1.2
cDBECPDPzP	1083.0 ± 1.2	28.9 ± 1.1	28.6 ± 1.2	164.5 ± 1.2	47.9 ± 1.1	548.8 ± 1.2
DBECPDPzP	941.9 ± 1.2	34.1 ± 1.1	31.7 ± 1.2	177.8 ± 1.2	64.2 ± 1.1	363.4 ± 1.1
DBECPDTP	201.7 ± 1.3	33.5 ± 1.1	37.2 ± 1.1	166.7 ± 1.3	42.1 ± 1.1	372.2 ± 1.1

## Supplementary Materials

Supplementary File 1
