# Supplementary material for: In Silico Study, Synthesis, and Cytotoxic Activities of Porphyrin Derivatives
Source: Pharmaceuticals (Basel). 2018 Jan 20;11(1):8. doi: 10.3390/ph11010008 (PMC5874704; doi:10.3390/ph11010008)
Supplement: Supplementary File 1 [file pharmaceuticals-11-00008-s001.pdf]

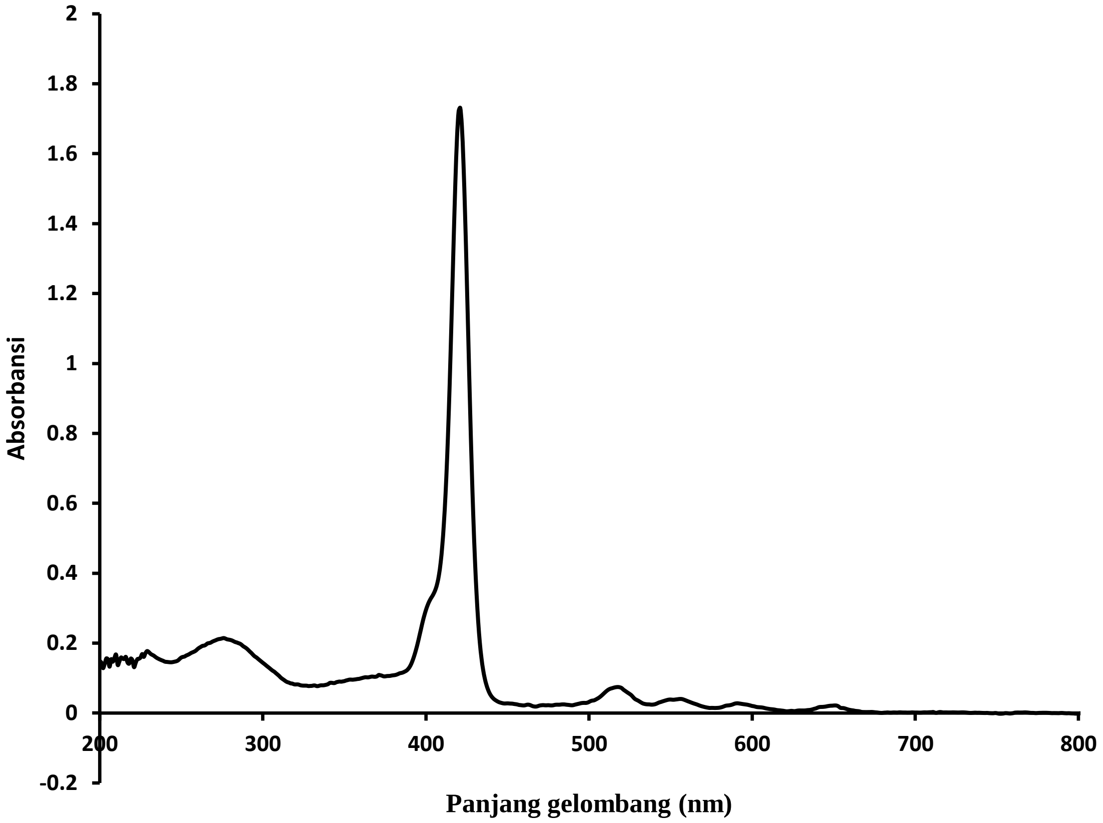

S1. UV/Vis spectra of DBECPDTP in CH_2Cl_2.

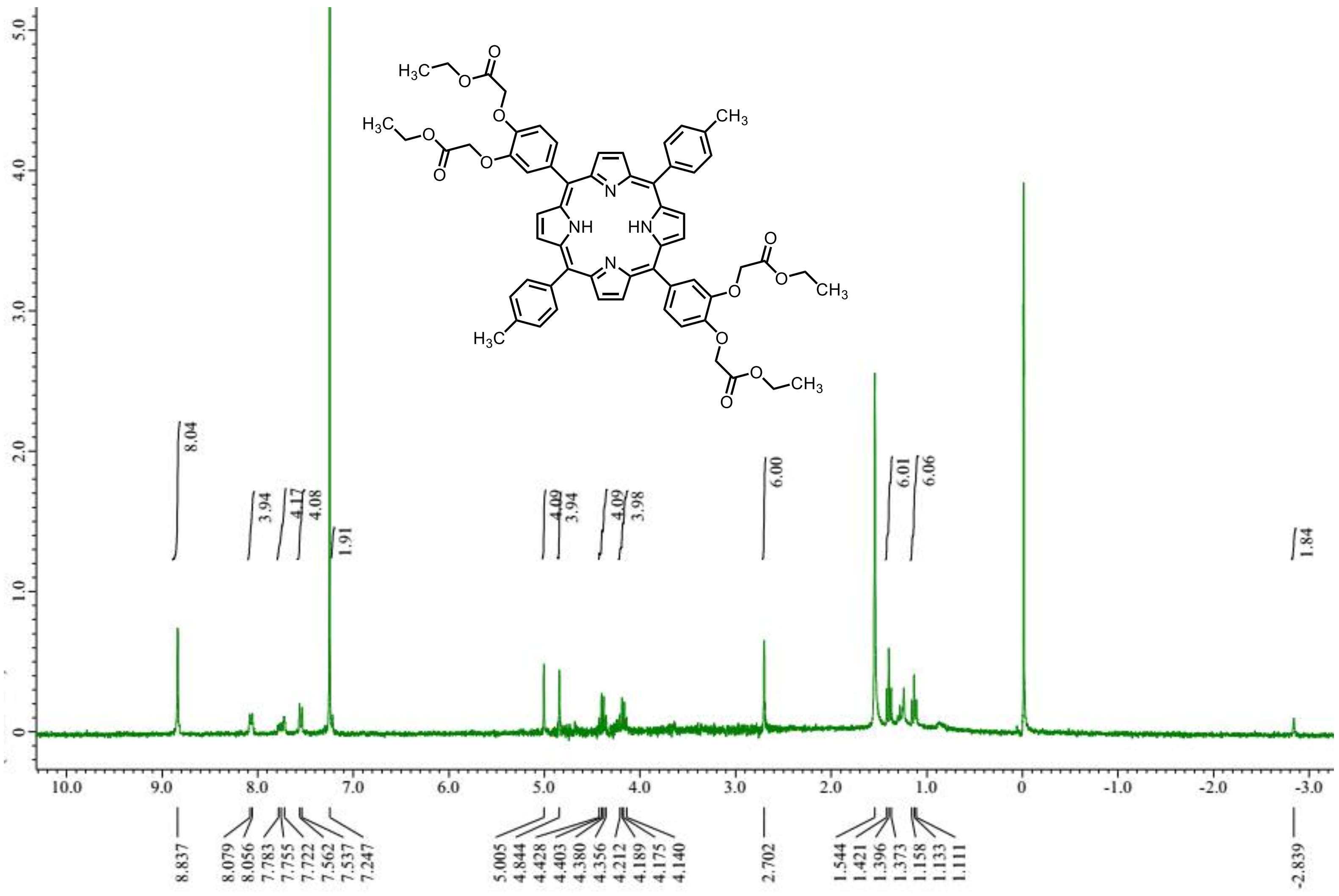

S2. ¹H-NMR ($CDCl_3$, 300 MHz) of DBECPDTP.

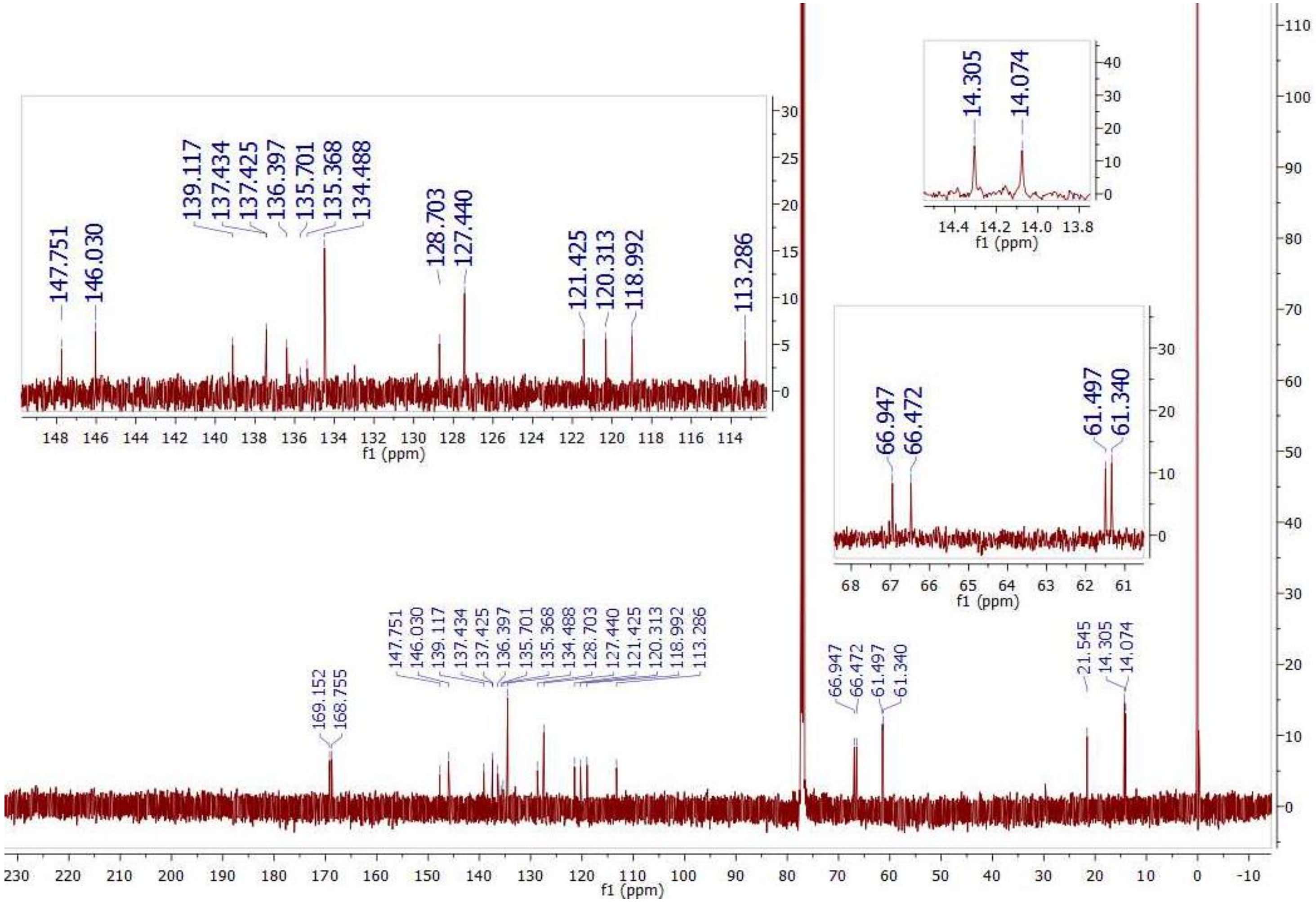

S3. ^{13}C-NMR ($CDCl_3$, 125 MHz) of DBECPDTP.

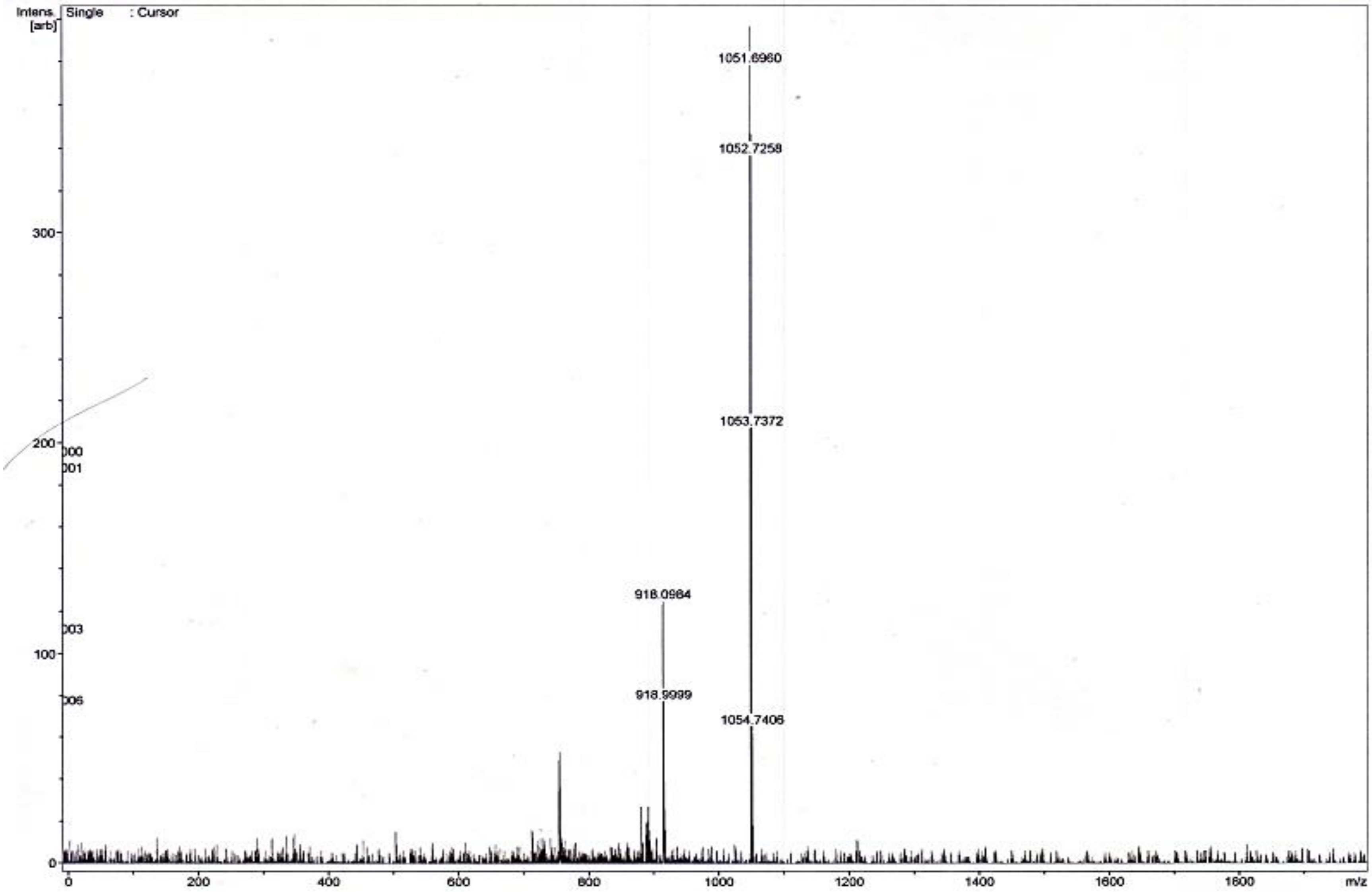

S4. MALDI-TOF LRMS of DBECPDTP.

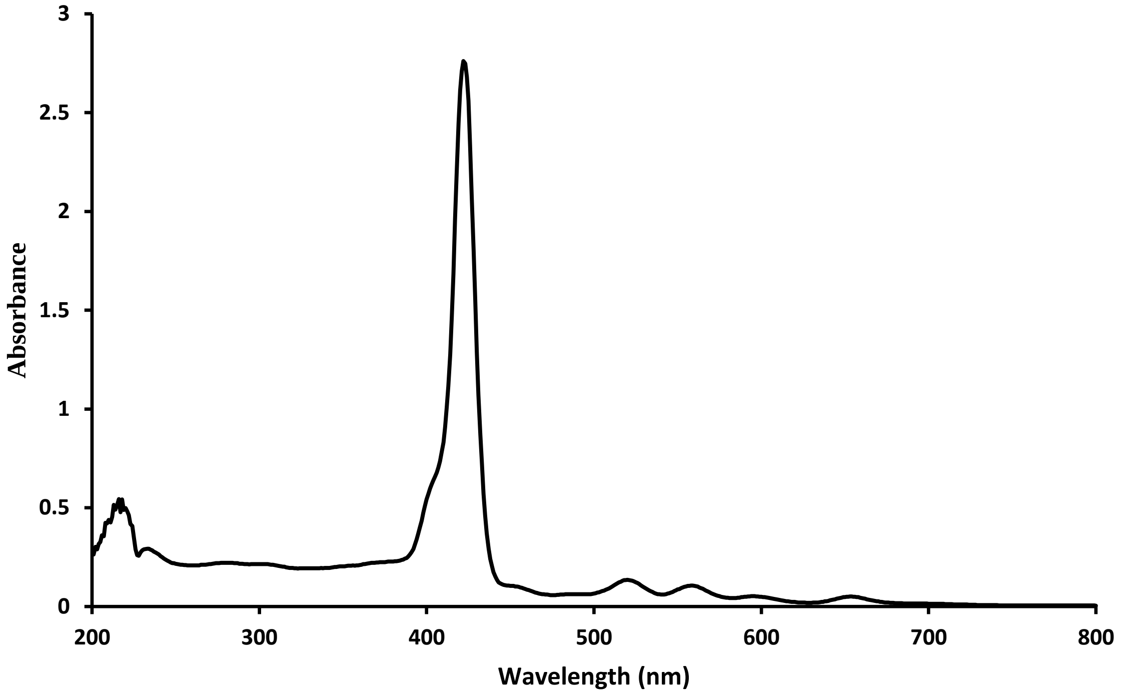

S5. UV/Vis spectra of cDBECPDPzP in CH_2Cl_2.

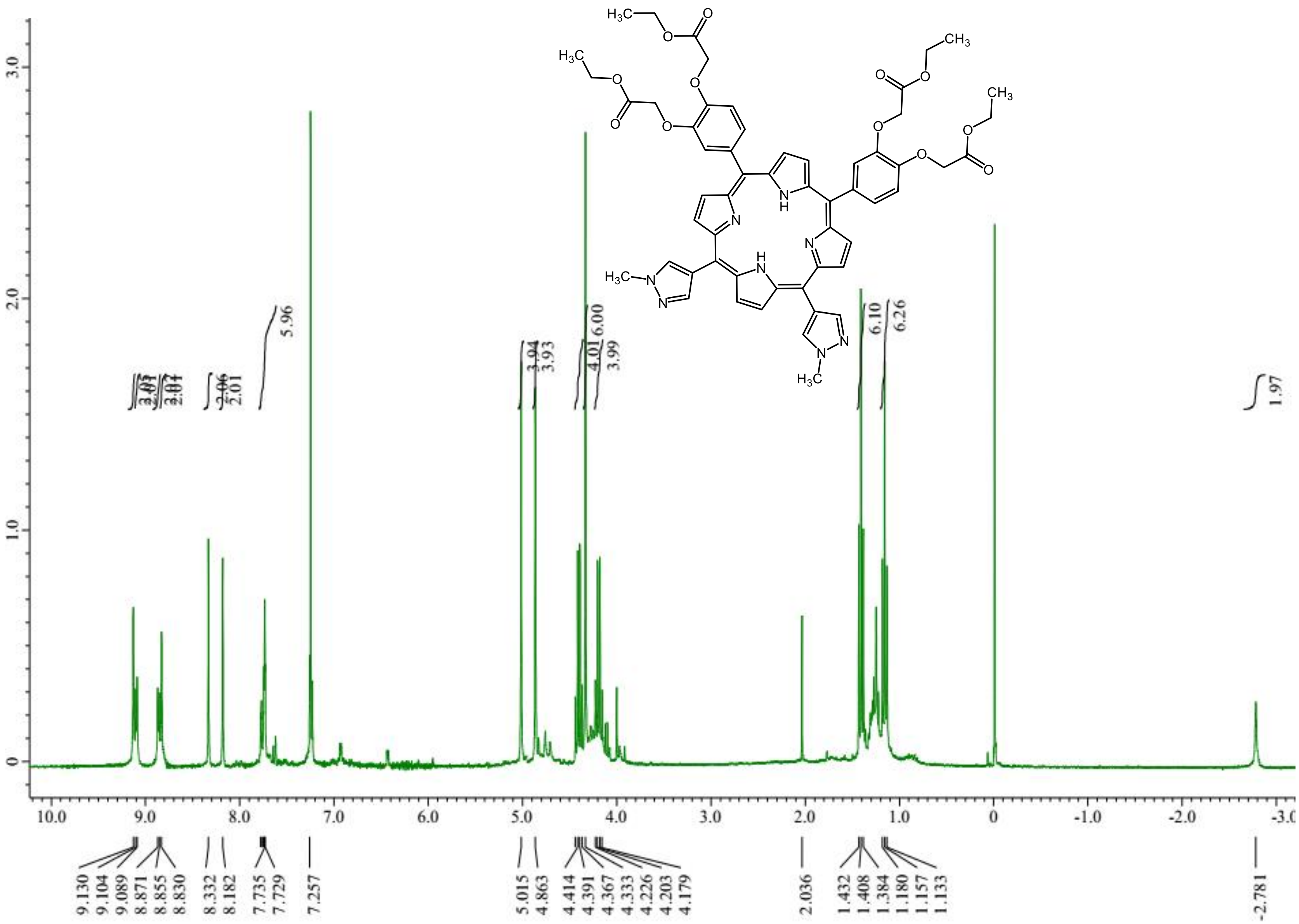

S6. ¹H-NMR ($CDCl_3$, 300 MHz) of cDBECPDPzP.

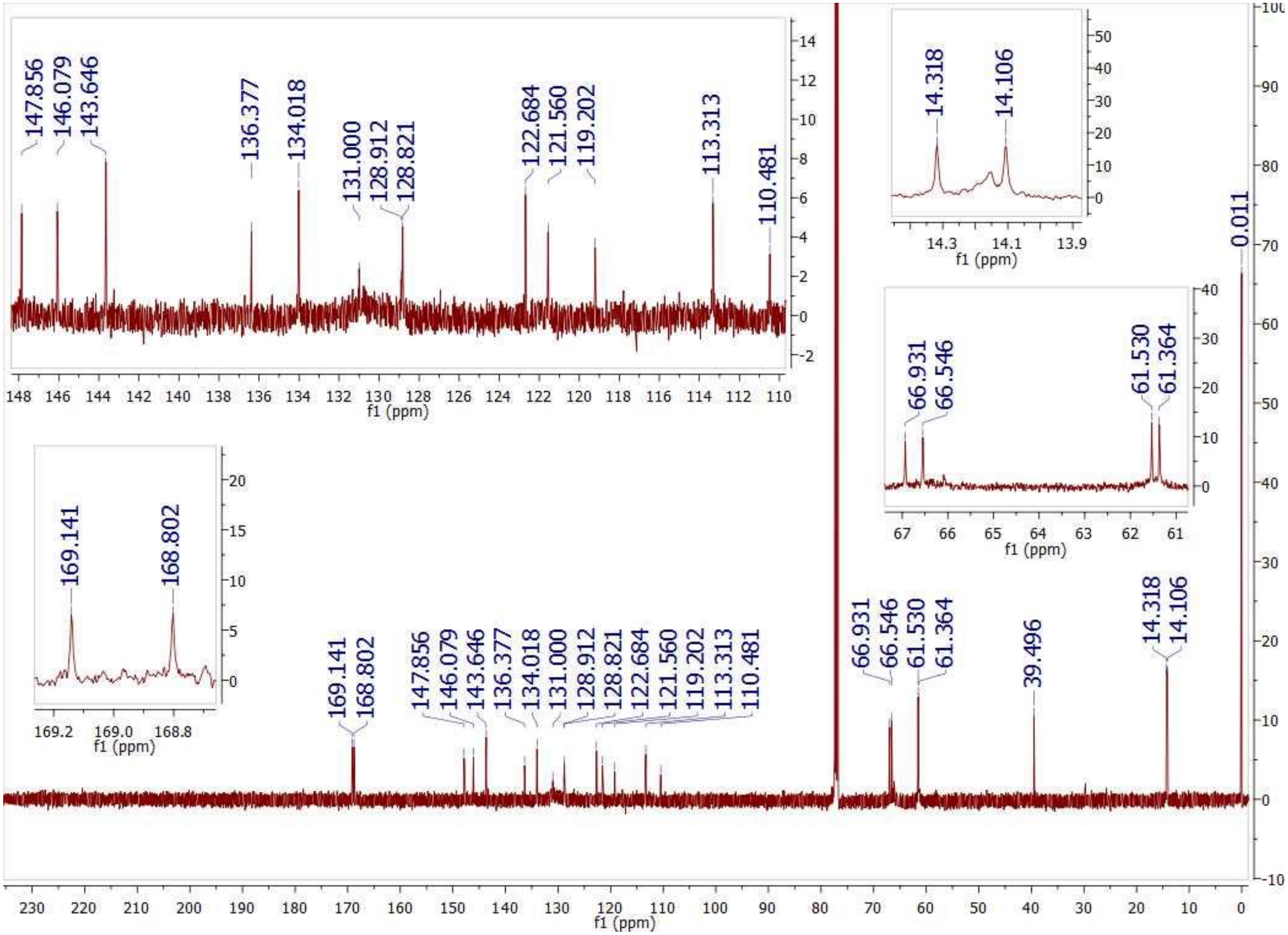

S7. ^{13}C-NMR ($CDCl_3$, 125 MHz) of cDBECPDPzP.

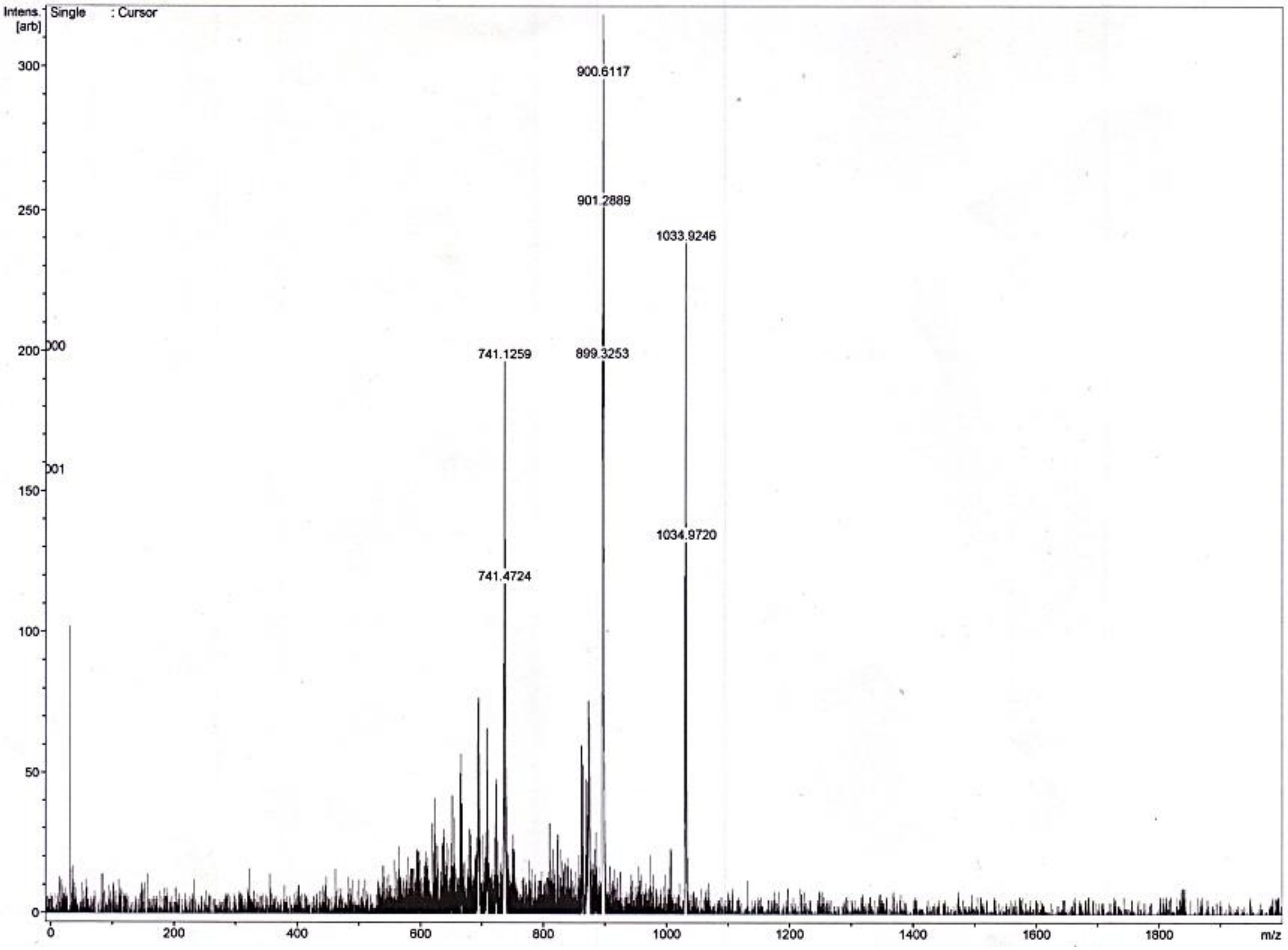

S8. MALDI-TOF LRMS of cDBECPDPzP.

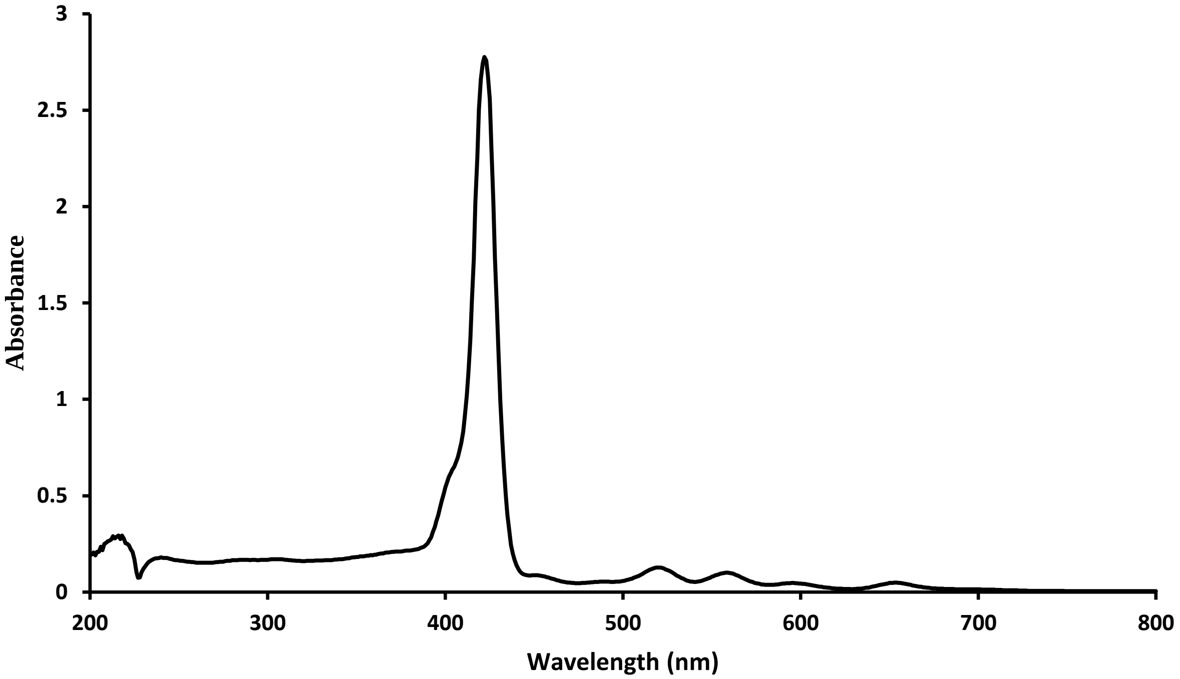

S9. UV/Vis spectra of DBECPDPzP in CH_2Cl_2.

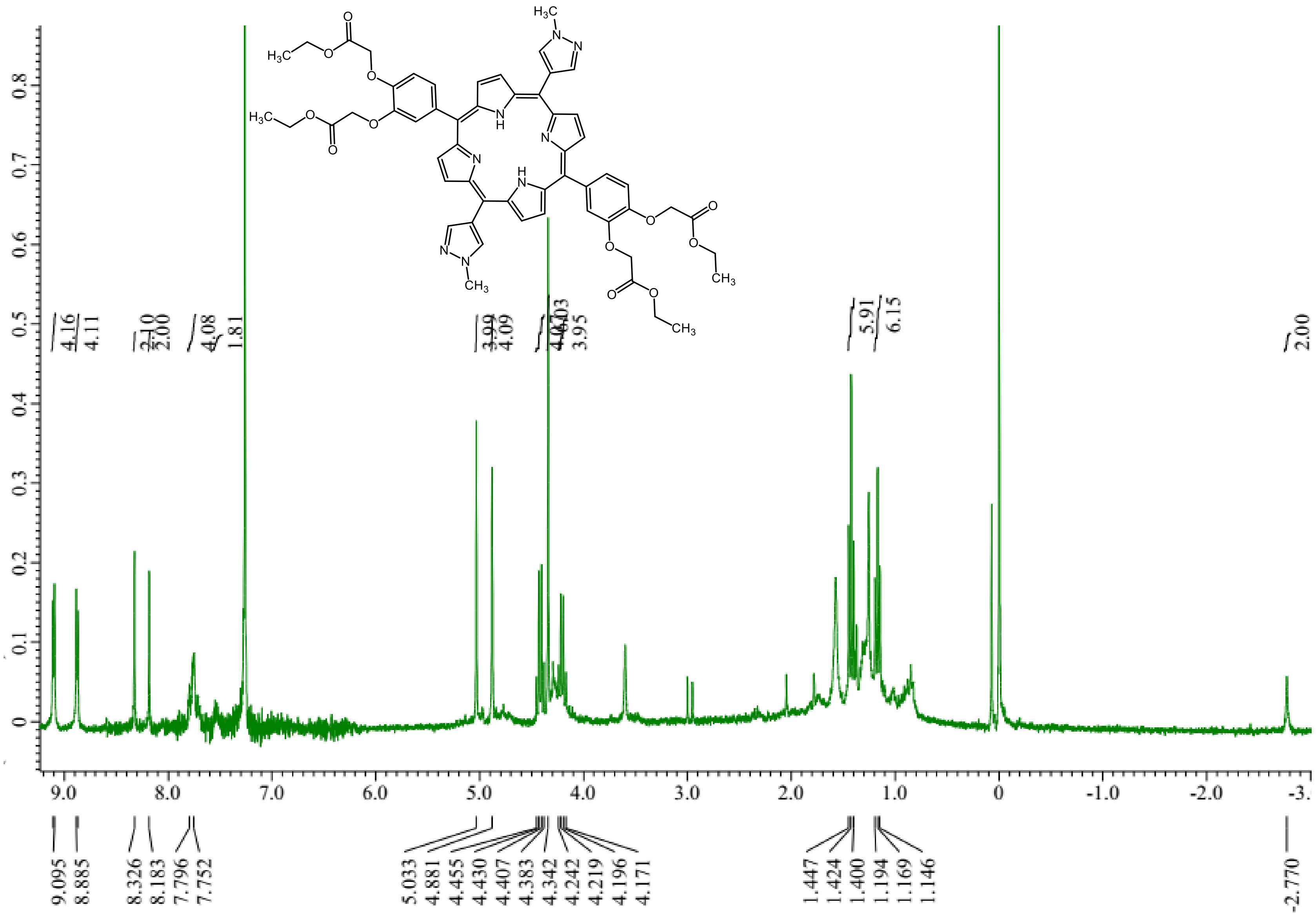

S10. ¹H-NMR ($CDCl_3$, 300 MHz) of DBECPDPzP.

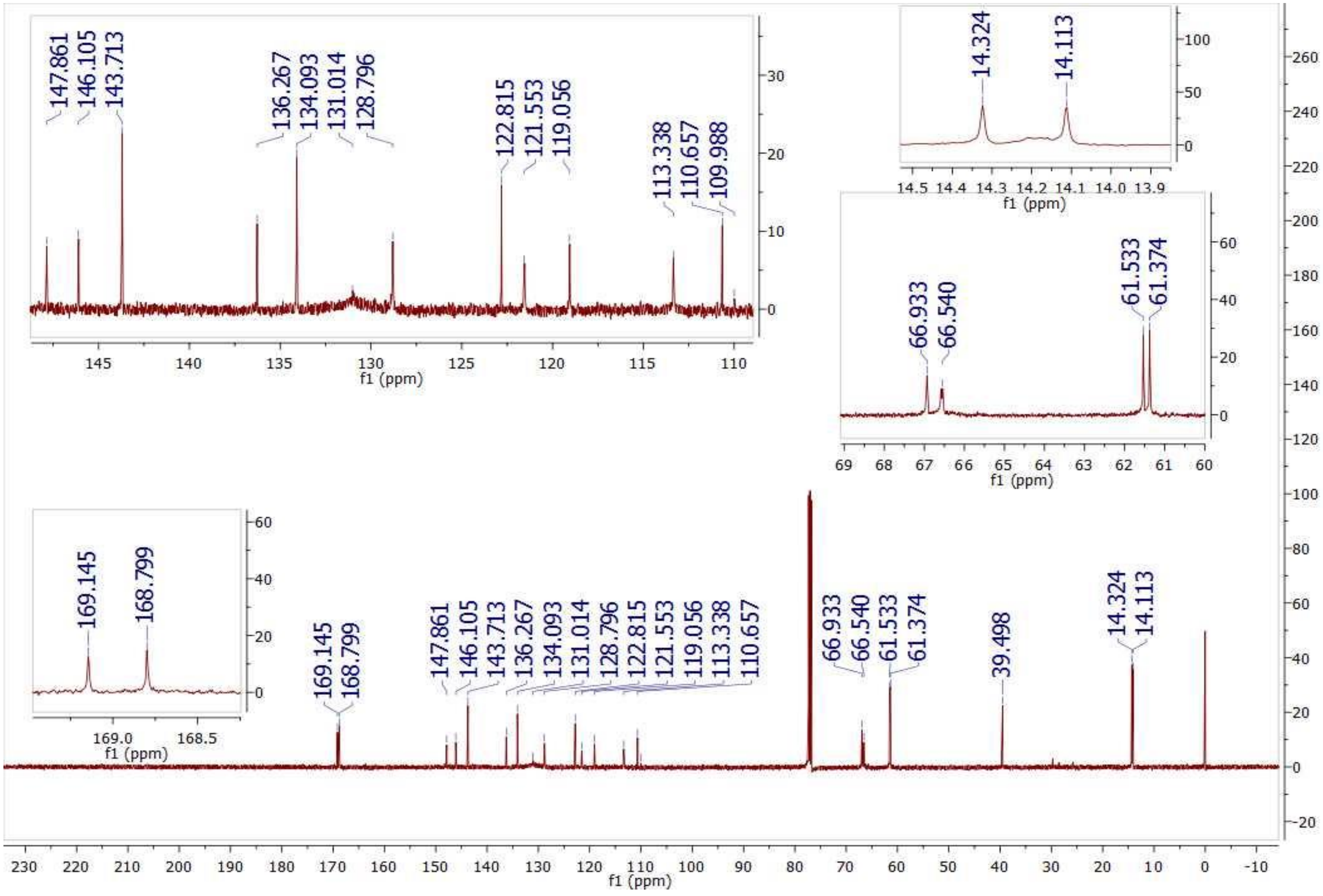

S11. ^{13}C-NMR ($CDCl_3$, 125 MHz) of DBECPDPzP.

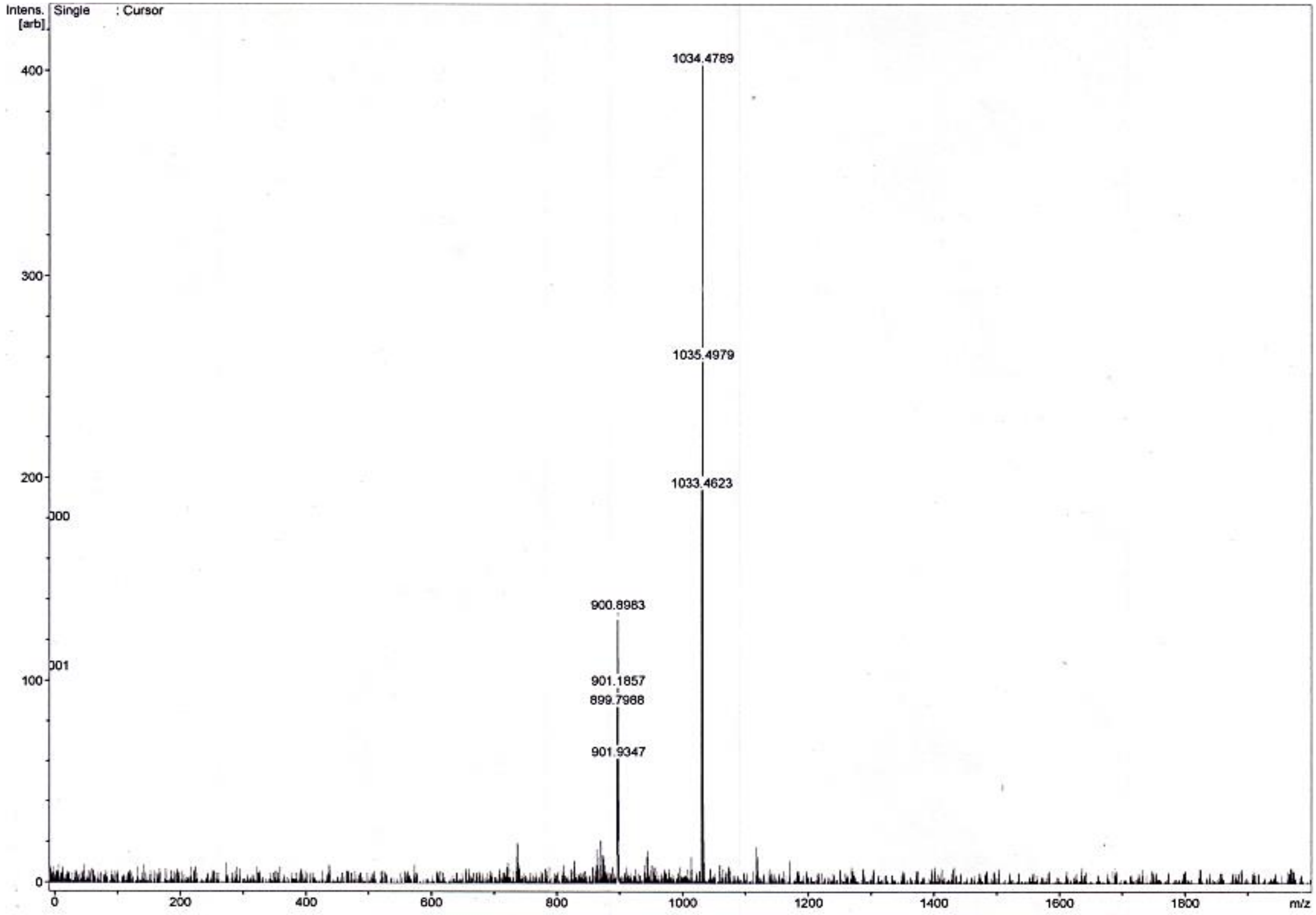

S12. MALDI-TOF LRMS of DBECPDPzP.
